# Intrauterine Smoke Exposure, microRNA Expression during Human Lung Development, and Childhood Asthma

**DOI:** 10.3390/ijms24097727

**Published:** 2023-04-23

**Authors:** Lynne Rosenberg, Cuining Liu, Rinku Sharma, Cheyret Wood, Carrie A. Vyhlidal, Roger Gaedigk, Alvin T. Kho, John P. Ziniti, Juan C. Celedón, Kelan G. Tantisira, Scott T. Weiss, Michael J. McGeachie, Katerina Kechris, Sunita Sharma

**Affiliations:** 1 Department of Pediatrics and Department of Medicine, University of Colorado Anschutz Medical Campus, Aurora, CO 80045, USA; 2 Department of Biostatistics and Informatics, Colorado School of Public Health, University of Colorado-Anschutz Medical Campus, Aurora, CO 80045, USA; 3 Channing Division of Network Medicine, Brigham and Women’s Hospital and Harvard Medical School, Boston, MA 02115, USA; 4 Children’s Mercy Hospital and Clinics, Kansas City, MO 64108, USA; 5 Division of Pediatric Pulmonary Medicine, University of Pittsburgh School of Medicine, Pittsburgh, PA 15261, USA; 6 Division of Pediatric Respiratory Medicine, Rady Children’s Hospital, University of California, San Diego, CA 92123, USA; 7 Division of Pulmonary Sciences and Critical Care Medicine, University of Colorado Anschutz Medical Campus, Aurora, CO 80045, USA

**Keywords:** allergy, asthma, gene expression, lung development, microRNAs, sex-specific

## Abstract

Intrauterine smoke (IUS) exposure during early childhood has been associated with a number of negative health consequences, including reduced lung function and asthma susceptibility. The biological mechanisms underlying these associations have not been established. MicroRNAs regulate the expression of numerous genes involved in lung development. Thus, investigation of the impact of IUS on miRNA expression during human lung development may elucidate the impact of IUS on post-natal respiratory outcomes. We sought to investigate the effect of IUS exposure on miRNA expression during early lung development. We hypothesized that miRNA–mRNA networks are dysregulated by IUS during human lung development and that these miRNAs may be associated with future risk of asthma and allergy. Human fetal lung samples from a prenatal tissue retrieval program were tested for differential miRNA expression with IUS exposure (measured using placental cotinine concentration). RNA was extracted and miRNA-sequencing was performed. We performed differential expression using IUS exposure, with covariate adjustment. We also considered the above model with an additional sex-by-IUS interaction term, allowing IUS effects to differ by male and female samples. Using paired gene expression profiles, we created sex-stratified miRNA–mRNA correlation networks predictive of IUS using DIABLO. We additionally evaluated whether miRNAs were associated with asthma and allergy outcomes in a cohort of childhood asthma. We profiled pseudoglandular lung miRNA in *n* = 298 samples, 139 (47%) of which had evidence of IUS exposure. Of 515 miRNAs, 25 were significantly associated with intrauterine smoke exposure (q-value < 0.10). The IUS associated miRNAs were correlated with well-known asthma genes (e.g., ORM1-Like Protein 3, *ORDML3*) and enriched in disease-relevant pathways (oxidative stress). Eleven IUS-miRNAs were also correlated with clinical measures (e.g., Immunoglobulin E andlungfunction) in children with asthma, further supporting their likely disease relevance. Lastly, we found substantial differences in IUS effects by sex, finding 95 significant IUS-miRNAs in male samples, but only four miRNAs in female samples. The miRNA–mRNA correlation networks were predictive of IUS (AUC = 0.78 in males and 0.86 in females) and suggested that IUS-miRNAs are involved in regulation of disease-relevant genes (e.g., A disintegrin and metalloproteinase domain 19 (*ADAM19)*, LBH regulator of WNT signaling (*LBH)*) and sex hormone signaling (Coactivator associated methyltransferase 1(*CARM1)*). Our study demonstrated differential expression of miRNAs by IUS during early prenatal human lung development, which may be modified by sex. Based on their gene targets and correlation to clinical asthma and atopy outcomes, these IUS-miRNAs may be relevant for subsequent allergy and asthma risk. Our study provides insight into the impact of IUS in human fetal lung transcriptional networks and on the developmental origins of asthma and allergic disorders.

## 1. Introduction

The World Health Organization reported that there were 1.3 billion tobacco users worldwide in 2021 (WHO 2021). In spite of the epidemiological data demonstrating poor health outcomes associated with smoking during pregnancy, the Centers for Disease Control and Prevention (CDC) reports that in 2016 roughly 7% of pregnant women in the United States smoked during pregnancy (CDC 2018), with some state-specific rates as high as 30%. Intrauterine smoke (IUS) exposure has been associated with myriad negative health consequences to the unborn child, including intrauterine growth restriction [1] and low birth weight [2]. Substantial epidemiologic data have also demonstrated that IUS exposure is associated with poor respiratory outcomes and allergic sensitization in the postnatal period, including reduced lung function [3,4,5], atopy [6,7,8], allergic sensitization to house dust mites [9], and susceptibility to later development of asthma [10,11,12,13,14,15].

Despite minimal human data elucidating the biological mechanisms underlying the epidemiologic association of IUS exposure with respiratory and allergic outcomes, there are ample animal data that demonstrate intrauterine-smoke-induced changes during lung development, which lead to increased postnatal disease susceptibility. In animal studies, maternal smoke exposure in the later stages of pregnancy has been associated with a multitude of adverse lung effects for the offspring, including fewer alveoli identified on lung histology [16], increased airway collagen deposition, lung hypoplasia [17], and proximal airway obstruction [18,19]. These observations supported the fetal origins hypothesis that intrauterine exposure may result in changes in fetal development that increase the risk of disease after birth. Importantly, these epidemiologic and animal studies motivate further investigation into how common intrauterine environmental perturbations, including toxic insults such as intrauterine smoke exposure, have the potential to affect the subsequent development of disease [20].

While the pathophysiology of IUS on fetal lung tissue is not entirely known, genomic profiling has started to reveal its complex impact [21]. Specifically, the changes in microRNA (miRNA) between IUS-exposed and unexposed lung tissue are of interest, given the role of miRNAs in the regulation of developmental processes. MicroRNAs are small, non-coding RNA molecules that are roughly twenty-two nucleotides in length and that regulate messenger RNA (mRNA) levels [22]. These molecules have been shown to have an important role in cell growth and apoptosis [23], development [24], differentiation [25], and disease [26,27,28,29,30]. Throughout development, miRNA expression profiles differ, depending on the stage of lung development [31,32]. In animal models, over- or under-expression of these miRNAs can affect multiple biological processes, eventually causing impaired lung development and the later development of lung disease, including asthma [33]. While cigarette smoking in adults has a miRNA signature [34], and while IUS within animal models also results in miRNA changes and subsequent lung dysfunction [35], there are a paucity of human data examining the impact of IUS in the fetal lung.

We aimed to better understand the effect of IUS exposure on miRNA expression in humans during the critical lung developmental period, hypothesizing that IUS-associated miRNAs may have regulatory impacts on pathways relevant to asthma and allergy. Using paired miRNA-sequencing and gene expression assays from a unique resource of human fetal lung tissue samples from the pseudoglandular stage of lung development—the time during which branching morphogenesis and airway development occur—we demonstrated that IUS is associated with changes in miRNA profiles and that the IUS-effect may be sex-specific. Furthermore, IUS-associated miRNAs are plausibly disease-related, based on their predicted mRNA targets, which are known to impact disease susceptibility, and their associations with clinical outcomes in a cohort of children with asthma.

## 2. Results

### 2.1. Sample Characteristics

We profiled miRNA and mRNA levels in *n* = 298 samples, *n* = 139 (47%) of which had evidence of IUS exposure, based on our validated assay measuring placental cotinine concentration (>7.5 ng/g) [36]; and *n* = 159 (53%) of which did not have evidence of IUS exposure. The distributions of estimated sample ages (days post-conception) and sex (male/female) were approximately equal in the two exposure groups (Table 1).

### 2.2. Differential Expression of miRNAs by IUS

After quality-control, 515 miRNAs were tested for differential expression using a dichotomized IUS-exposure group. After adjusting for age, sex, run, and RUVr factors, we detected 25 miRNAs significantly associated with IUS (q-value < 0.10; Table 2). Interestingly, the IUS-miRNAs were previously reported in studies of smoke exposure, including changes in let-7 and miR-10 family miRNAs between smokers versus non-smokers in the human airway, as well as in a mouse lung model of IUS (Supplementary File S1, Supplementary Figure S1).

Possible empirical mRNA targets for each of these miRNAs, measured by pairwise correlation to fetal lung microarray data, are provided in Table 2 and include genes critical to both lung development (e.g., angiogenesis genes *VEGFC*, *DLL4*) and asthma and allergy *(CXCL16*, *ORDML3*, *IL4R*, *ILF3)*.

### 2.3. Pathway Enrichment Analysis

These possible functional consequences were also echoed in pathway analyses based on the theoretical mRNA targets: IUS-miRNAs were enriched in lung development pathways such as Wnt signaling [37] and branching morphogenesis. Importantly, there was also enrichment in pathways associated with environmental stressors, including oxidative stress response and the metabotropic glutamate receptor pathway.

### 2.4. Sex-Specific Associations between IUS and miRNAs

**Figure 1:**
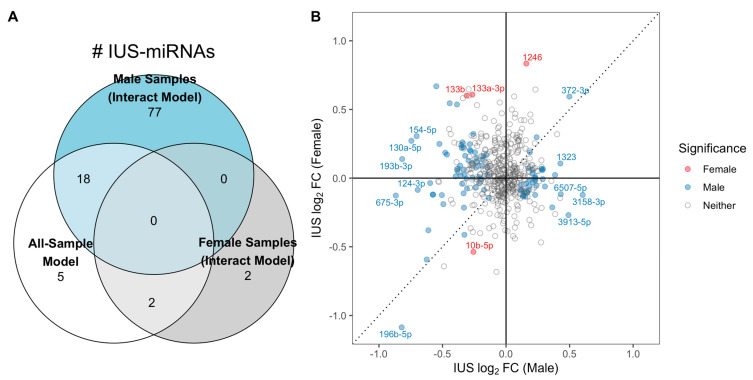
Evidence for sex-specific miRNA-IUS in fetal lung. (**A**) Number of significant IUS-miRNAs detected per model. A greater number of IUS-miRNAs were detected when allowing for sex-specific effects using the sex-by-IUS interaction model, particularly among males: 95 IUS-miRNAs were detected only among males, with only 18 of the 95 detected previously in the all-sample no interaction model. In contrast, only four total IUS-miRNAs were detected in female samples, and 0 of these 4 were significant among male samples. (**B**) Effect estimates were poorly correlated between males and females. Each point represents one miRNA’s mean, covariate-adjusted log2-fold change associated with IUS exposure in male (*x*-axis) and female (*y*-axis) fetal lung, colored according to which groups had significance in the interaction model. The effects have limited correlation, with many miRNA switching effect directions between sexes (top-left and bottom-right quadrants; e.g., hsa-miR-133b abundances increased with IUS in females but decreased with IUS in males).

Given the known evidence of sexual dimorphism of miRNA expression during development [35,38] and sex-specific differences in asthma and atopy [39,40], we repeated the statistical modeling procedure allowing for sex-specific IUS effects. We found substantial differences in IUS effects by sex: of the 515 miRNAs tested, 110 miRNAs had significantly different IUS effect estimates by sex (i.e., IUS-by-sex interaction q-value < 0.10; results tables and stratified miRNA–mRNA empirical correlations in Supplementary File S2, Table S1), the majority of which (99 of 110) came from autosomal chromosomes. We detected 95 miRNAs significantly associated with IUS in male samples (i.e., non-zero IUS effect, q-value < 0.10) (Table S2), whereas only four miRNAs were significantly associated with IUS in female samples (non-zero IUS effect, q-value < 0.10) (Table S3). There was little overlap between the set of significant male IUS-miRNAs and female IUS-miRNAs (Figure 1A), and effect estimates were poorly correlated between sexes (Spearman r = −0.11, Figure 1B).

### 2.5. Sex-Specific miRNA–mRNA Networks of IUS

**Figure 2:**
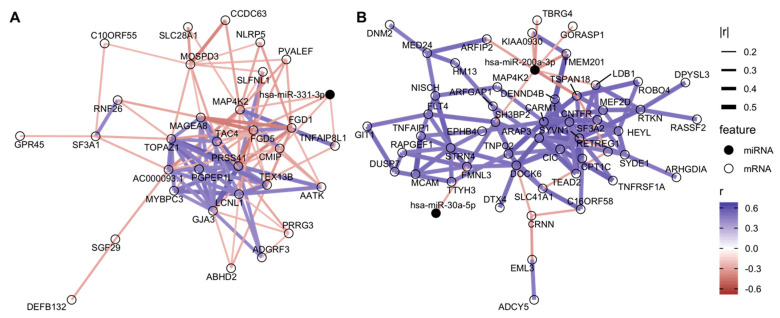
Sparse sex-specific miRNA–mRNA networks associated with IUS. The features selected from DIABLO were used to generate correlation networks, with edges representing between-feature Spearman correlations. For visualization, we show only edges with high between-feature correlation and nodes with at least one “large” correlation (r < −0.25 or r > 0.40 based on 10th and 90th quantile values; full list of features in Supplementary File S3). This process was performed separately on (**A**) male samples and (**B**) female samples. For example, in female fetal lung, *hsa-miR-200a-3p* was empirically negatively correlated with clinically relevant genes such as *TSPAN18* and *ARFIP2*, which were in turn positively correlated with a larger set of genes, including *CARM1*, *DENND4B*, and *HEYL*.

We applied DIABLO to our dataset, to identify miRNA and mRNA features both correlated across the ‘omes and discriminatory of IUS-exposures. The male-only DIABLO model selected one miRNA, *hsa-miR-331-3p*, significantly decreased with IUS in males (log_2_FC = −0.46 based on interaction model) and negatively correlated with genes such as *NLRP5* and *FGD5*; DIABLO correlation networks in Figure 2A. In females, *hsa-miR-29c-3p* and *hsa-miR-200a-3p* were selected (Figure 2B). Interestingly, many miRNA-gene correlations in the female samples were weaker in male samples and vice versa, further supporting a potential sex-specific impact of IUS on miRNA abundances and regulation patterns (Supplementary File S3, Supplementary Figures S2–S5). For example, the correlation between *miR-200a-3p* and *TBRG4* was −0.29 (female-only) versus −0.10 (all samples). Only one mRNA (*MAP4K2*) was selected in both models. The two miRNAs decreased with IUS-exposure in female fetal lung samples were not found to be significant in the univariate model (respective IUS log_2_FC = −0.59 and −0.15 in female samples, q-values = 0.17 and 0.18), yet the DIABLO analysis generated models with high discriminative ability between IUS-exposed and unexposed samples (AUC = 0.78 in males and 0.86 in females), suggesting that prioritization of miRNAs with strong mRNA correlations could identify additional miRNAs for further study.

### 2.6. Impact of IUS-Associated MicroRNAs on Asthma and Allergy Outcomes in a Childhood Asthma Cohort

Lastly, we tested for associations between the IUS-miRNAs identified in the all-samples analysis and clinical outcomes in an independent cohort of children with asthma (GACRS) and with serum miRNA-seq profiles. Of the 25 IUS-miRNAs significant in the all-sample analysis, 17 were detected. Eleven demonstrated differential expression with markers of asthma severity and atopy in childhood asthmatics (Table 3). Notably miR-101-3p, miR-10b-5p, miR140-3p, miR19b-3p, and miR-543 were each associated with both asthma and atopy. One miRNA (miR-423-5p) was only associated with markers of atopy, including increased immunoglobulin E and house dust mite sensitization (HDM). Five IUS-associated miRNAs were associated with lung function, but not markers of atopy in the childhood asthmatics. Importantly, IUS exposure decreased miR-200a-3p expression in human fetal lung, was identified in the female miRNA–mRNA network of IUS, and was associated with decreased ratio of forced expiratory volume in one second/ forced vital capacity (FEV_1_/FVC) in childhood asthmatics. Repeating these analyses stratifying GARCS by gender, we found that some of these miRNAs may have sex-specific associations with asthma and allergy outcomes: for example, after stratification, associations between miR-101-3p and clinical outcomes were only detected in the male subcohort (Table 4).

## 3. Discussion

Despite the preponderance of evidence demonstrating the substantial negative health consequences of smoking, maternal smoking during pregnancy remains a large public health problem. Epidemiological data have demonstrated that exposure to intrauterine smoke is associated with asthma and allergic sensitization in childhood [11,12] and has a long-lasting impact, with demonstrably lower lung function in adults exposed to IUS [41]. Given the critical role that miRNAs play in the regulation of gene expression during lung development, an understanding of the impact of IUS exposure on miRNA expression is essential for elucidating the impact of IUS exposure on the biological processes of lung development and subsequent asthma and allergy susceptibility. By measuring miRNA-seq, gene expression assays, and placental cotinine concentrations with a unique human fetal lung dataset, we contribute new evidence about the potential developmental origins of asthma and atopy. Our study suggests that IUS exposure alters miRNA profiles during early pseudoglandular lung development and that these miRNAs likely influence biological pathways associated with later disease susceptibility, due to their associations with disease-relevant pathways, target genes, and clinical measures of asthma and atopy in a cohort of childhood asthmatics.

The pseudoglandular period is the histological stage of lung development during which branching morphogenesis and airway development commence [42]. Biological processes that are fine-tuned by miRNAs during this important developmental stage are essential for normal airway development [43]. In addition, regulation of immune cell function through miRNA and other epigenetic mechanisms during the early stages of lung development has been shown to also influence allergic sensitization [44]. Thus, understanding how IUS exposure impacts the miRNA expression signature is important for understanding how this common intrauterine perturbation impacts subsequent susceptibility to asthma and allergy. Using miRNA-sequencing in human fetal lung tissue from the pseudoglandular stage of lung development, we identified 25 miRNAs that were differentially expressed with IUS exposure in all samples. Based on databases of mRNA targets (miRWalk), the genes regulated by the IUS-miRNAs are involved in airway development and biological pathways relevant to environmental exposures, including oxidative stress response and the metabotropic glutamate receptor pathway, which are highly relevant to asthma and allergy pathobiology.

In concordance with our findings, previous investigations also suggested that regulation of genes involved in the oxidative stress response in early lung development has critical implications for allergy and asthma susceptibility in the postnatal period. The developing fetus is highly vulnerable to oxidative stress injury, as both the immune system and lung structural cells are immature and highly susceptible to toxic exposure, even during these early developmental stages [45,46]. The increased oxidative burden in subjects exposed to IUS activates inflammatory cells and results in an imbalance between oxidants and antioxidants, resulting in direct damage to components of the lung matrix [47]. Oxidative stress also results in increased apoptosis, mucus hypersecretion, and decreased binding affinity and translocation of steroid receptors [47]. Moreover, IUS exposure has been shown to delay the maturation of the immune system and alter both innate and adaptive immune responses in offspring, leaving the offspring highly susceptible to postnatal infection [48]. Notably, oxidative stress mechanisms have been implicated in the development of asthma, low lung function, and allergy [49].

Our results also suggest that IUS results in dysregulation of the metabotropic glutamate receptor (mGLuR) pathway, which is also relevant to the development of asthma and allergy. The mGLuRs are members of the G-protein coupled receptor family [50]. These receptors have been identified as slow regulators of glutamate transmission [51]. While this pathway is extensively involved in neuronal signaling, these receptors are also expressed in the airway and are thought to play a role in bronchodilation and airway remodeling [52]. Several of these receptors have been implicated in nicotine addiction and may be targets for smoking cessation pharmacotherapies in the future [53]. While our results suggest that IUS may alter these plausible disease-associated pathways in a miRNA-mediated fashion, validation studies assessing how the candidate IUS-miRNAs alter functional measures such as oxidative burden and airway remodeling are crucial, to causally link prenatal miRNA expression dysregulation to post-natal disease.

As post-natal phenotypic profiling of disease features is not possible with fetal tissue, examining IUS–miRNA correlates with clinical outcomes in relevant cohorts is one of the few options available for functional evaluation within human tissues and in vivo. While we recognize that this may limit the clinical utility of our findings, our findings demonstrate biological relevance. With the caveat that our findings are associative and not causal, and that the miRNA impacts could be context-specific (e.g., some targets expressed in prenatal airway formation only), we investigated whether the miRNAs differentially expressed by IUS were associated with lung function and markers of atopy in children with asthma. Of the 17 IUS-associated miRNAs that were available for evaluation in GACRS, we found that 11 of them demonstrated an association with lung function and/or atopy in a well-characterized cohort of childhood asthmatics, offering further support for our hypothesis that IUS-associated miRNAs may be important in the biological mechanisms underlying the development of asthma and allergy.

For example, we found that IUS exposure was associated with increased miR-543 expression in human fetal lung tissue. Based on our empirical miRNA–mRNA correlations, miR-543 regulates the intrauterine expression of several genes implicated in atopy, including the endoplasmic reticulum aminopeptidase 2 (*ERAP2*) gene. Genetic polymorphisms in *ERAP2* have been previously associated with atopic dermatitis risk, suggesting a potential role in the development of atopy [54]. We advanced our previous understanding of this complex biology by demonstrating the association of miR-543 with other markers of atopy, including house dust mite sensitization, peripheral eosinophil counts, and immunoglobulin E levels in GACRS. Notably, increased expression of miR-543 was associated with decreased markers of atopy in childhood asthmatics, suggesting that IUS may resulted in decreased atopic sensitization. While the role of miR-543 in allergic responses has not been tested directly, He et al. previously described an association of miR-543 with the development and progression of COPD, compared to healthy controls [55]. While increased miR-543 was found in COPD subjects, it was also shown to regulate disease progression through interleukin 33 (*IL-33*) signaling [55]. *IL-33* plays an important role in type-2 innate immunity, via activation of allergic inflammation-related eosinophils [56]. While these findings suggest that miR-543 may drive atopic sensitization via *IL-33*, additional investigation will be needed to confirm this finding.

Similarly, we showed that miR-19b-3p is decreased with IUS exposure in human fetal lung tissue and empirically correlated with two genes implicated in allergenic inflammation within both humans and animal models: *SPRR2* [57] and *TLR3* [58]. In childhood asthmatics, this is associated with increased atopy (IgE level, peripheral blood eosinophils), increased FEV_1_/FVC, and decreased bronchodilator response. These findings mirror previous work in asthma, in which Bersimbaev et al. found that plasma levels of hsa-miR-19b-3p were downregulated in patients with bronchial asthma and asthma-COPD overlap syndrome, and upregulated in chronic obstructive pulmonary disease [59]. Additional investigation of this miRNA is warranted.

We additionally found evidence for sex-specificity in IUS-exposure associations with miRNA expression. By allowing for sex-by-IUS interactions in regression modeling, we found 95 miRNAs significantly associated with IUS in male samples, whereas only four miRNAs were significantly associated with IUS in female samples. For example, miR-101-3p significantly decreased with IUS-exposure in our all-sample analysis and in male samples, but not in female fetal lung samples. We additionally showed that decreased expression of miR-101-3p is associated with increased house dust mite sensitization and increased bronchodilator response in childhood asthmatics, with this finding again detected in all-sample and male subjects, but not female subjects. To our knowledge, this miRNA has not previously been examined in a sex dimorphism context, but its clinical relevance is corroborated by findings from Wang and colleagues in a mixed-gender adult asthma study. Wang et al. found that miR-101-3p was downregulated in asthmatic subjects as compared to healthy controls and that miR-101-3p expression was correlated with interleukin 13 (*IL-13*) levels, measures of pulmonary function, and increased inflammation [60]. The authors also demonstrated that miR-101-3p is differentially expressed between asthmatic and healthy subjects after rhinovirus inoculation, suggesting a role for miR-101-3p in immune response, through targeting interferon induced with helicase C domain 1 (*IFIH1*), which has been shown to sense rhinoviruses. They postulate that miR-101-3p may play a regulatory role in rhinovirus-induced asthma [60].

Support for the sex-specific impacts of IUS on miRNA profiles was also evident in our sex-stratified miRNA–mRNA network analyses. Using DIABLO, we discovered sparse candidate regulatory networks that had strong miRNA–mRNA correlations, while being highly discriminative of IUS exposure (predicting exposures with cross-validation AUC = 0.78 in males and 0.86 in females). Strikingly, no miRNAs and only one mRNA gene were common between the male-only model and the female-only networks, and the miRNA–mRNA correlations found in one sex tended to be much lower in magnitude in the other sex. However, both networks involved genes plausibly relevant to allergy, airway remodeling, and disease. The male-only DIABLO model selected one miRNA, *hsa-miR-331-3p* (significantly decreased with IUS only in male fetal lung) and mRNAs such as the Wnt-signaling regulator *LBH*, *FLRT1*, *RNF26*, and *SLFNL1*. In the female-only model, *hsa-miR-29c-3p* and *hsa-miR-200a-3p* were selected and negatively correlated with genes of biological relevance, including *ADAM19*, *AGO1*, *CRNN*, and *SLC41A1* [61,62]. Furthermore, the gene present in both models was *MAP4K2*; this gene family has been extensively associated with airway inflammation in asthma and COPD, including experimental demonstrations that it can be induced by cigarette smoke exposure [63]. We also noted genes associated with sex hormone pathways within the sex-specific networks: CARM1, for example, is putatively involved in estrogen signaling [64] and was a major hub gene in the female-only model. This analysis thus suggests that sex-specific IUS-effects might be partly driven by hormonal differences, and more broadly associates IUS-exposure with transcriptional networks including known disease genes. The features in this network can be prioritized as future targets for mechanistic studies of IUS exposure and fetal lung development and disease susceptibility.

Given that the IUS-miRNAs for both sexes are plausibly associated with asthma and allergy susceptibility (based on empirical mRNA correlations, GARCS, and existing literature), our work contributes a better understanding of miRNAs and gene targets, to prioritize functional investigation into the poorly understood mechanisms underlying IUS exposure and possible post-natal disease risks. Our work adds to reports of the potential sex-specific impacts of IUS [65]. We thus suggest considering sex as a critical effect-modifier in future studies of IUS and/or the developmental origins of asthma and atrophy, particularly as sex-specific differences in clinical disease outcomes are also well known [39,40].

Our study has several limitations. Our prenatal lung samples were obtained from a de-identified fetal tissue biorepository, so only sample age, IUS, and sex were available. We attempted to address potential unmeasured confounders such as fetal comorbidities and other environmental exposures through statistical approaches (RUV), but residual confounding may still have been present. We also note that our measure of intrauterine smoke exposure (placental cotinine concentration) does not preclude exposure to nicotine via other methods of consumption or it may not capture effects due to cumulative smoking habits (maternal pack-years). Similarly, our study may have underestimated the transcriptional dysregulation due to cumulative IUS exposure, and many epidemiological studies often find third-trimester IUS to be the most predictive of future disease risk; however, we found striking shifts in miRNA profiles even within very early lung development, in samples with gestational ages from the first and early second trimester of pregnancy. Lastly, validation is challenging for studies of the developmental origins of disease. Technical replication was not possible, due to the limited quantity of RNA available from these rare tissues, and functional validation was not performed. Although our results suggest that IUS-associated miRNAs are associated with asthma and allergy risk based on mRNA targets and comparison to clinical outcomes in childhood asthmatics, future studies should use animal or cell-line models, to measure the functional impact of the miRNAs and whether those impacts persist from pre-natal life to early childhood.

## 4. Materials and Methods

### 4.1. Fetal Lung Sample Acquisition and Metadata

Human fetal lung tissue samples were collected as part of a prenatal tissue retrieval program sponsored by the National Institute of National Child Health and Development (NICHD), the University of Maryland Brain and Tissue Bank for Developmental Disorders (Baltimore, MD, USA), and the Center for Birth Defects Research (University of Washington; Seattle, WA, USA), as previously described [37]. The study was designated an institutional review board (IRB) exempt protocol by the University of Missouri-Kansas City Pediatric IRB, Partners Human Research Committee IRB, and the Colorado Multiple Institutional Review Board (COMIRB).

Due to de-identification, only estimated post-gestational age, IUS, and sex were available for each sample. We assessed IUS with placental cotinine concentration (Cotinine Direct ELISA kit, Calbiotech, Spring Valley, CA, USA), using an assay our group previously demonstrated had high sensitivity and specificity (AUC = 0.88), relative to self-reported maternal cigarette smoking status [36]. The samples are a subset of previously published work profiling human fetal lung gene expression [66], in which we classified sample sex as male or female based on their expression of X- and Y-chromosome genes in Affymetrix Human Gene 1.0 ST microarrays.

### 4.2. miRNA Profiling

Our methods for fetal lung miRNA profiling and analysis are described in detail within a previous publication [38]. Briefly, we extracted total RNA from 30 mg of homogenized prenatal lung tissue (Qiagen miRNeasy Mini Kit; Valencia, CA, USA). Samples were randomized to four plates for miRNA library preparation (Small RNA Sequencing Kit v3 for Illumina Platforms; Bioo Scientific, Austin, TX, USA) and sequencing (Illumina HiSeq2500; San Diego, CA, USA). Reads were trimmed with cutadapt [67], then mapped using miR-MaGiC [68] with reference to the miRbase v22.1 database. Quality-control was applied to samples (≥1 × 10^5^ mapped reads, no missing metadata, age 7–17 weeks pseudoglandular period), and miRNAs (non-zero counts in at least 25% of samples).

### 4.3. Differential miRNA Levels by IUS

We regressed the count of each miRNA (outcome) in the IUS group (dichotomized at exposed at ≥ or non-exposed at <7.5 ng cotinine/g placenta) in the DESeq2 negative binomial model, adjusting for post-conceptional age and age^2^ (quadratic term to capture potential non-linear effects), sex (male or female), technical batch (indicator variable for library plate 1, 2, 3, or 4), and four inferred covariates (k = 4 RUVr components [69] representing unmeasured sources of miRNA expression heterogeneity; *k* selected based on the elbow method, with the total percentage of miRNA variance explained by RUV plateauing at 4 components) for inclusion in regression models. A statistically significant difference in mean miRNA levels by IUS was defined using a Wald test at a prespecified multiple testing corrected Storey q-value of <0.10; that is, we tolerated up to 10% false positives among our reported IUS-miRNAs. The pre-specified value of 10% was chosen because it is commonly used, as it provides a reasonable margin of error for genomics studies.

### 4.4. Sex-Specific Effects of IUS

We also considered the above model with an additional sex-by-IUS interaction term, allowing IUS effects to differ between male and female samples. We tested for statistically significant interactions (Wald test, q-value < 0.10), indicative of miRNAs with sex-specific IUS effects, and within in each sex tested if their IUS effect significantly differed from the null (Wald test, q-value < 0.10).

### 4.5. Predicted mRNA Targets and Pathway Analyses

A central hypothesis of our work was that IUS-miRNAs may be associated with known asthma and allergy-related mRNA targets. Thus, as one approach to interpreting the functional impact of IUS-miRNAs, we examined the hypothesized mRNA targets of each miRNA (collected in miRWalk databases of in silico binding site prediction and miRNA–mRNA binding assays) and performed a GSEA pathway analysis using the miEAA 2.0 webportal [70] applied to miRWalk pathways (Benjamini–Hochberg FDR < 0.10) [71].

### 4.6. miRNA–mRNA Correlations

A limitation of target predictions is that miRNA–mRNA correlations may be context-specific. The aforementioned paired microarray measurements allowed us to calculate empirical correlations between IUS-miRNAs and mRNA gene targets within the fetal lungs. We first residualized both miRNA and microarray probe intensities for covariates, then examined pairwise miRNA–mRNA Spearman correlations. The intention of residualization is to remove technical noise (e.g., batch effects specific to each dataset) and sample metadata: namely, global miRNA and mRNA profiles strongly vary with post-conceptional age in this dataset [38]. Thus, naïve correlations may reflect broad age-miRNA and age-mRNA associations. We thus residualized both features for specificity towards putative miRNA–mRNA regulatory patterns, albeit with the trade-off of potentially under-reporting miRNA–mRNA correlations. The miRNA levels were represented by applying the variance stabilizing transformation (VST) to miRNA counts, then regressing out all covariates but IUS from the primary model (age, age^2^, sex, batch, and RUV components). Similarly, mRNA levels were represented using log_2_ (probeset intensities) and regressed on age, age^2^, sex, batch, and RUVr components (k = 4, recalculated separately for microarray data).

To compliment these pairwise correlation tests, we used the multivariate, multi-omic (i.e., considers multiple miRNAs and mRNAs at a time) integration algorithm DIABLO [72] to integrate the residualized datasets and perform feature selection, to identify the top candidate miRNA–mRNA networks discriminatory of IUS status. Given the evidence of sex-specific IUS miRNA signatures, DIABLO was run separately on male samples and female samples. Sensitivity analyses were performed to examine sex-specificity and robustness to model parameters (details in Supplementary File S3).

### 4.7. IUS-miRNAs Associations with Asthma and Allergy Outcomes in a Childhood Asthma Cohort

As a final assessment of the hypothesis that IUS-associated miRNAs may regulate biological pathways that are relevant to asthma and allergy, we evaluated whether IUS-miRNAs were associated with asthma and allergy outcomes in a well-characterized cohort of childhood asthma. The Genetics of Asthma in Costa Rica Study (GACRS) is a cross-sectional study of 1165 Costa Rican children with asthma aged 6 to 14 years and recruited between February 2001 and August 2008. A previous publication [73] described the GACRS protocols and assessments. Clinical outcomes used here were as follows: forced expiratory volume in one second as a percent predicted (FEV_1_PP), dichotomized as the mean value; the ratio of FEV_1_ to forced vital capacity (FEV_1_/FVC), dichotomized as the mean value; bronchodilator response (BDR), measured as a percent change in FEV_1_ dichotomized at 8% [74]; the log10 transformed immunoglobulin E (IGE), dichotomized as the geometric mean value; peripheral eosinophils (EOS), dichotomized at 300 cells/µL; and sensitization to Derp 1 or house dust mite (HDM) using a skin prick test [73].

Small RNA sequencing (RNA-seq) was performed on the serum samples from 1134 GACRS children following established protocols [75]. Small RNA-seq libraries were prepared with a Norgen Biotek Small RNA Library Prep Kit (Norgen Biotek, Therold, Canada) sequenced on the Illumina NextSeq 500 platform. The ExceRpt pipeline was utilized for quality control and mapping [76]. miRNAs detected (≥5 counts) in at least 50% of GACRS subjects and significantly differentially expressed with IUS exposure in human fetal lung were tested for differential expression with respect to the above dichotomized clinical outcomes in GACRS, adjusting for age and gender using DESeq2 (FDR < 0.10). A gender-stratified analysis for these miRNAs was also performed.

### 4.8. Study Reproducibility

Analysis code (github.com/chooliu/FetalLung_miRNA_SmokeExposure, accessed on 19 March 2023) and raw/processed miRNA-sequencing and microarray data for fetal lung are publicly available (NCBI GEO deposition GSE200153, GSE8896).

## 5. Conclusions

In summary, we identified differential expression of miRNAs by IUS exposure in human lung development, that these miRNAs as associated with well-known genes and biological pathways associated with asthma and atopy, and that these miRNAs correlated with clinical asthma and atopy measures in a well-characterized cohort of childhood asthma. Our work provides support for the growing body of evidence that miRNAs are critical to normal lung development and that common intrauterine exposures such as maternal smoking can impact lung development in a sex-specific manner and influence subsequent disease susceptibility. These data provide additional support for the developmental origin of asthma and allergy.

## Figures and Tables

**Table 1:** Sample Characteristics.

	No IUS Exposure	IUS-Exposure	All Samples
N	159	139	298
Age (dpc) *	87.0 (76.0, 98.0)	89.0 (76.0, 96.0)	87.0 (76.0, 96.0)
Placental Cotinine (ng/g)	0.0 (0.0, 0.8)	53.5 (27.1, 79.6)	2.2 (0.0, 50.4)
Sex (Male)	89 (56.0%)	77 (55.4%)	166 (55.7%)

* Age and placental cotinine values are presented by the median (interquantile range) and sex is count (percent of total column). Dpc is defined as days post conception.

**Table 2:** Differentially Expressed miRNAs by IUS Exposure in Human Fetal Lung Development.

	miRNA	log_2_FC	lfSE	q-Val	Top Microarray Correlations
Increases with IUS	hsa-miR-372-3p	0.54	0.16	0.031	VEGFC^+^, SLC27A6^+^, KRTAP20-2^+^, BACH2^+^, CYP4F2^+^, EHD2^+^, TOMM40L^+^, POMGNT2^+^
	hsa-miR-1246	0.45	0.15	0.059	PTTG1^+^, HIST1H2BG^+^, USP34^−^, CNBP^+^, HIST2H2BE^+^, SUOX^−^, CYP51A1^+^, YTHDF3^−^
	hsa-miR-1323	0.28	0.07	0.003	GNPAT^+^, PSMD13^+^, ORMDL3^+^, CAMLG^+^, ELOA^+^, FBXO5^+^, SERPINB1^+^, MDH1^+^
	hsa-miR-431-5p	0.27	0.08	0.031	MUC1^+^, PNMA5^−^, SELENBP1^+^, KDM1B^+^, SERPINB2^−^, SLCO2B1^+^, CXCL16^+^, HIST1H3E^−^
	hsa-miR-320d	0.19	0.07	0.059	OTUD7A^−^, INSYN2A^+^, ADCY5^+^, ARSD^+^, AMN1^+^, AOC3^+^, NAALADL1^+^, BAIAP2L1^+^
	hsa-miR-3613-5p	0.18	0.07	0.072	MCAM^+^, ABCF2^+^, SREK1IP1^−^, TTC38^+^, SLC25A11^+^, PRPS1^+^, RCL1^+^, NMT2^+^
	hsa-miR-543	0.17	0.06	0.099	TDRD12^+^, ERAP2^−^, OR2M5^−^, ACSL4^−^, AREG^−^, HCRTR2^−^, MAP7^−^, RBM11^−^
	hsa-miR-423-5p	0.16	0.05	0.031	CLOCK^+^, ENTPD7^+^, JRK^−^, IBA57^+^, LTV1^+^, MYL3^+^, CMSS1^+^, ADCY5^+^
	hsa-let-7e-5p	0.14	0.04	0.036	GLMN^+^, ELSPBP1^−^, ZNF45^−^, CRH^−^, KCNH5^−^, INPP5A^+^, PDE8A^+^, CCDC9B^+^
Decreases with IUS	hsa-miR-148a-3p	−0.12	0.04	0.059	HIST1H2BH^−^, OR52N2^+^, CATSPER3^+^, HIST1H2AL^−^, MMP16^−^, HIST1H2BO^−^, FOXC1^−^, HIST1H2BJ^−^
	hsa-miR-200a-3p	−0.13	0.04	0.050	PPP1R1A^−^, CCL2^−^, AKIRIN1^−^, PXK^−^, DGLUCY^−^, SNAI1^−^, S100A3^+^, GPR148^+^
	hsa-miR-101-3p	−0.14	0.04	0.040	EXOC7^−^, FRRS1L^+^, CAD^−^, NFATC1^−^, MTOR^−^, PPP4C^−^, WDR76^−^, CEP85L^−^
	hsa-miR-125b-5p	−0.17	0.05	0.040	UBE4B^−^, VPS39^−^, KIF20A^−^, NCAPD2^−^, ST3GAL6^−^, PDLIM1^−^, FOXM1^−^, KLHDC3^−^
	hsa-miR-532-5p	−0.17	0.06	0.056	EIF4G1^−^, SIPA1L2^−^, TRIM8^−^, NUP98^−^, ILF3^−^, RASGEF1A^+^, MTOR^−^, TTLL5^−^
	hsa-miR-221-5p	−0.17	0.07	0.092	KHSRP^−^, KIRREL1^−^, PFAS^−^, SEC24D^−^, FMNL3^−^, ADGRA2^−^, SPTLC1^+^, EHD2^−^
	hsa-miR-324-5p	−0.17	0.06	0.076	CHRNA1^−^, TSPAN15^−^, KCNN2^−^, ALDH18A1^−^, CHGB^−^, IL4R^−^, DCTN5^−^, PSMD13^−^
	hsa-miR-584-5p	−0.19	0.07	0.059	DHRS9^+^, DLL4^−^, DHX32^+^, SAP130^−^, SDSL^+^, DAB2IP^−^, GABPA^+^, AGO1^−^
	hsa-miR-34a-5p	−0.19	0.06	0.040	CDH7^−^, SCGB1D4^+^, GCN1^−^, THY1^−^, PSG4^+^, MRPL20^−^, MRPL20^−^, LZIC^+^
	hsa-miR-19b-3p	−0.21	0.06	0.020	CGA^+^, GCNT3^+^, SPRR2E^+^, TREH^+^, NAT8^+^, TLR3^+^, OR10S1^+^, CUEDC1^−^
	hsa-miR-140-3p	−0.37	0.11	0.031	ENTPD7^−^, CYP19A1^−^, OGT^+^, FMN1^+^, NR4A2^+^, B3GNT5^−^, LIPG^−^, FAM20A^−^
	hsa-miR-10b-5p	−0.38	0.11	0.026	STAB2^+^, PROX1^+^, RELN^+^, CCL21^+^, SMPD4^−^, IRS4^−^, PDE2A^+^, CGA^−^
	hsa-miR-29a-3p	−0.51	0.11	0.003	BTG4^+^, DLK1^−^, ALDH8A1^+^, PCSK4^−^, C4BPB^+^, AKAP4^+^, EDN3^+^, GML^+^
	hsa-miR-675-3p	−0.54	0.18	0.051	KRTAP10^−^3^+^, ZNF263^−^, KRTAP10-12^+^, ASTN2^+^, FAM78B^+^, POLR2E^−^, TAF8^−^, BLVRB^−^
	hsa-miR-29c-3p	−0.60	0.16	0.020	TRAF3IP2^−^, GJA4^−^, STX5^−^, TOM1^−^, TBC1D17^−^, PCIF1^−^, FTL^−^, DPF2^−^
	hsa-miR-196b-5p	−0.93	0.26	0.031	UTS2R^−^, NOP10^−^, ALDH16A1^+^, OR14C36^+^, ORM1^−^, SFMBT1^+^, UBL5^+^, GNPAT^−^

The twenty-five miRNAs that significantly differed (DESeq2, q-value < 0.10) by intrauterine smoke exposure are shown, with effect estimate (log_2_FC and standard error), q-value, and top eight correlations with genes in paired microarray data. The direction of correlation is shown as a superscript: for example, *hsa-miR-431-5p* is positively correlated with *MUC1* and negatively correlated with *PNMA5*.

**Table 3:** Associations between miRNA Levels and Asthma/Allergy Outcomes in a Childhood Asthma Cohort (GACRS). We tested the hypothesis that miRNAs differentially expressed with IUS in fetal lung samples (Table 2) are associated with asthma and allergy relevant biological pathways, by evaluating their clinical associations in a cohort of childhood asthmatics. miRNA-outcome associations with FDR < 0.10 are shown.

miRNA	baseMean	log2FoldChange	FDR	Outcome *
hsa-miR-101-3p	36,355.424	−0.406	0.001828	HDM
	36,896.186	−0.255	0.045888	BDR
hsa-miR-10b-5p	208,777.388	0.287	6.97 × 10^7^	IGE
	209,492.165	0.234	0.000494	EOS
	208,991.420	0.183	0.023477	HDM
	208,591.796	0.296	1.07 × 10^6^	FEV_1_PP
	208,591.796	−0.133	0.027242	FEV_1_/FVC
	210,699.398	−0.247	0.000545	BDR
hsa-miR-140-3p	21,185.162	−0.564	5.61 × 10^5^	HDM
	21,724.957	−0.372	0.004762	BDR
hsa-miR-19b-3p	1272.550	0.507	0.002363	IGE
	1264.854	0.419	0.03691	EOS
	1282.529	0.590	0.00032	FEV_1_/FVC
	1284.974	−0.493	0.011979	BDR
hsa-miR-423-5p	120,957.129	−0.263	0.000638	IGE
	120,792.953	−0.318	0.000837	HDM
hsa-miR-543	124.481	−0.783	0.000494	EOS
	124.296	−0.928	0.000569	HDM
	124.448	−0.545	0.01383	IGE
	125.414	0.545	0.036505	BDR
hsa-miR-200a-3p	440.055	0.286	0.02454	FEV_1_/FVC
hsa-miR-221-5p	2126.747	0.285	0.006463	FEV_1_/FVC
hsa-miR-29a-3p	6308.506	0.354	0.00032	FEV_1_/FVC
hsa-miR-29c-3p	353.824	0.507	0.002031	FEV_1_/FVC
hsa-miR-532-5p	783.174	0.438	0.006463	FEV_1_/FVC

* HDM: house dust mite test (positive vs. negative), IGE: log_10_ serum immunoglobulin E (mean value cut-off), EOS: peripheral blood eosinophil raw count (300 cut-off), BDR: bronchodilator response (8% cut-off), FEV_1_PP: percent predicted FEV_1_ (mean value cut-off), FEV_1_/FVC: ratio of the forced expiratory volume in one second/forced vital capacity (mean value cut-off).

**Table 4:** Gender-Stratified Associations between miRNA Levels and Asthma/Allergy Outcomes in GACRS. Due to sex-specific IUS effects being detected in fetal lung, we repeated the miRNA-outcome association testing (Table 3) with gender stratification. Some miRNAs were uniquely observed in one gender.

miRNA	baseMean	log_2_FoldChange	*p*-Value	FDR	Outcome *
**Male**					
hsa-miR-101-3p	37,732.87	−0.69266	2.37 × 10^6^	1.5 × 10^5^	HDM
hsa-miR-101-3p	37,495.54	−0.48684	3.42 × 10^5^	0.000163	IGE
hsa-miR-10b-5p	221,947.5	−0.23555	0.004001	0.038013	BDR
hsa-miR-10b-5p	220,378.5	0.228005	0.003688	0.023355	EOS
hsa-miR-10b-5p	219,178.4	−0.23061	0.001313	0.012474	FEV_1_/FVC
hsa-miR-10b-5p	219,247	0.313008	1.52 × 10^5^	9.6 × 10^5^	IGE
hsa-miR-10b-5p	219,178.4	0.502512	2.26 × 10^11^	4.29 × 10^10^	FEV_1_PP
hsa-miR-140-3p	21,853.83	−0.98959	1.49 × 10^10^	2.83 × 10^9^	HDM
hsa-miR-140-3p	21,885.73	−0.70774	1.17 × 10^8^	2.23 × 10^3^	IGE
hsa-miR-140-3p	22,081.31	−0.41309	0.001861	0.017682	FEV_1_PP
hsa-miR-29a-3p	5927.324	−0.35357	0.001905	0.0181	EOS
hsa-miR-423-5p	130,667.4	−0.30672	0.000622	0.011822	FEV_1_/FVC
hsa-miR-423-5p	130,778.8	−0.5239	3.50 × 10^6^	1.66 × 10^5^	HDM
hsa-miR-423-5p	130,273.6	−0.43617	1.22 × 10^6^	1.16 × 10^5^	IGE
hsa-miR-543	142.4763	0.907437	0.000937	0.017796	BDR
hsa-miR-543	140.9837	−1.11925	2.20 × 10^5^	0.000418	EOS
hsa-miR-543	140.4037	−1.54768	4.05 × 10^7^	3.85 × 10^6^	HDM
hsa-miR-543	141.141	−0.97293	7.37 × 10^5^	0.00028	IGE
**Female**					
hsa-miR-148a-3p	13,627.06	0.325966	0.000904	0.01717	IGE
hsa-miR-1246	190.8938	-0.51509	0.001765	0.03354	FEV_1_/FVC

* HDM: house dust mite test (positive vs negative), IGE: log_10_ serum immunoglobulin E (mean value cut-off), EOS: peripheral blood eosinophil raw count (300 cut-off), BDR: bronchodilator response (8% cut-off), FEV_1_PP: percent predicted FEV_1_ (mean value cut-off), FEV_1_/FVC: ratio of the forced expiratory volume in one second/forced vital capacity (mean value cut-off).

## Data Availability

Analysis code (github.com/chooliu/FetalLung_miRNA_SmokeExposure, accessed on 19 March 2023) and raw/processed miRNA-sequencing and microarray data for fetal lung are publicly available (NCBI GEO deposition GSE200153, GSE8896).

## Supplementary Materials

The supporting information can be downloaded at: https://www.mdpi.com/article/10.3390/ijms24097727/s1.
