# Supplementary material for: Intrauterine Smoke Exposure, microRNA Expression during Human Lung Development, and Childhood Asthma
Source: Int J Mol Sci. 2023 Apr 23;24(9):7727. doi: 10.3390/ijms24097727 (PMC10178351; doi:10.3390/ijms24097727)
Supplement: Supplementary file 1 [file ijms-24-07727-s001.zip › ijms-2325380-supplementary.pdf]

Online Supplemental Materials

Intrauterine smoke exposure, microRNA expression during human lung development, and childhood asthma

<u>Supplementary File S1: Comparisons to Published miRNA Results</u>

<u>About:</u> We compared whether the miRNAs differentially expressed with IUS exposure in fetal lung (IUS-miRNAs) overlapped with miRNAs previously reported with maternal smoking or cigarette exposure/smoking history more broadly.

<u>Conclusions:</u> Seven fetal lung IUS-miRNAs (all-samples analysis, male samples, or female samples IUS-miRNA lists) were previously reported in at least one maternal smoking / smoker versus non-smoker study: miR-25-5p, miR-133b, miR-199a-3p, miR-423-5p, miR-449a, miR-543, and miR-1246.

Or, considering any overlap in miRNA families, 33 miRNA IUS-miRNA families have previously been reported: let-7, miR-10, miR-19, miR-24, miR-25, miR-26, miR-30, miR-34, miR-106, miR-124, miR-125, miR-126, miR-130, miR-133, miR-140, miR-145, miR-146, miR-190, miR-193, miR-199, miR-214, miR-224, miR-365, miR-374, miR-423, miR-449, miR-486, miR-500, miR-520, miR-543, miR-652, miR-675, and miR-1246.

Our study showed the highest fractional overlaps with Wang et al. (2015), Schembri et al. (2009), Huang, et al. (2019), Izzotti, et al. (2009), and Dehmel et al. (2018) which are respectively three human adult airway epithelium studies, one adult rat lung study, and one fetal lung intrauterine smoke exposure mouse study collected at E18.5 (**Figure S1**). Interestingly, the focus on pseudoglandular fetal lung samples correspond to airway formation and branching.

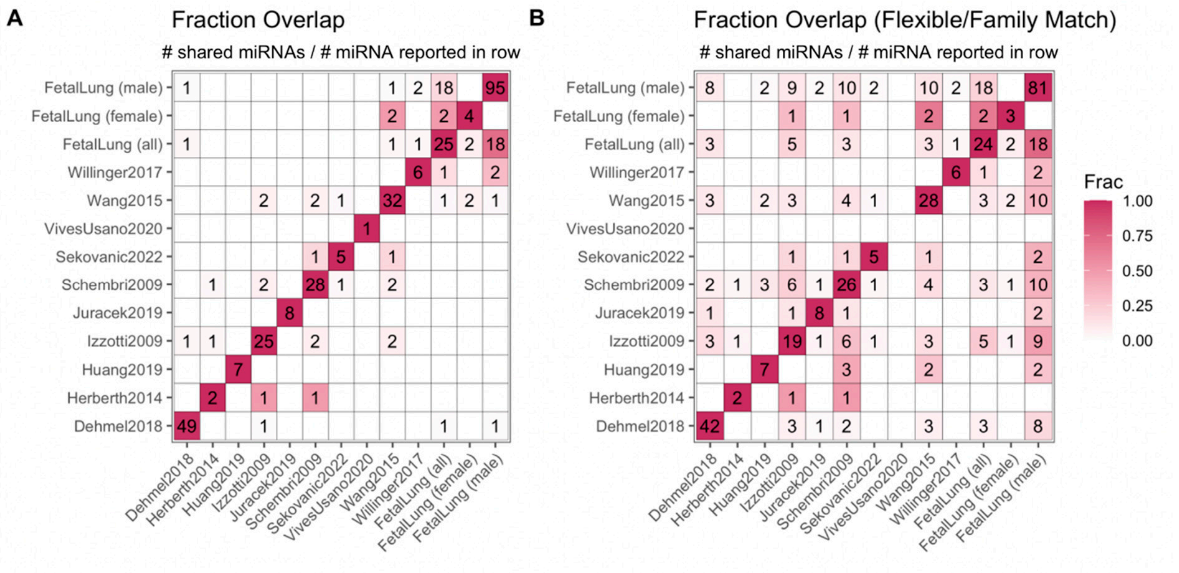

Supplementary Figure S1. The number in each tile shows the number of significant miRNAs reported by each study for **(A) exact matches** or **(B) miRNA family matches** (see methods below for details). The color of each tile shows the number of overlaps divided by the total number reported by the study listed in the row. For example, in Panel B, 8 miRNA families are reported both in the male fetal lung samples and Dehmel et al. (2018). The fraction is 8 / 81 = 0.099.

The direction of tobacco/smoking effect was not necessarily concordant (see next pages for details). This said, we observed limited overlap in exposure signatures across all studies examined. We found 13 miRNA families to be reported within three or more studies (including our fetal lung work): miR-10, miR-34, miR-106, miR-124, miR-125, miR-130, miR-145, miR-181, miR-199, miR-218, miR-223, miR-449, miR-500. Four or five studies reported miR-30, let-7, and miR-146.

Methods

Literature Review. We used the following search terms in NCBI/Pubmed and Google Scholar to systematically select up to 10 previously published papers for comparison on March 30th, 2023.

("miRNA" OR "microRNA") AND ("maternal smoking" OR "intrauterine smoke exposure")
("miRNA" OR "microRNA") AND (("prenatal" OR "in utero" OR "pregnancy") AND ("cigarette" OR "smoke" OR "smoking" OR "tobacco"))
("miRNA" OR "microRNA") AND ("lung" OR "airway" OR "alveolar") AND ("cigarette" OR "smoke" OR "smoker" OR "tobacco" OR "smoking")
("miRNA" OR "microRNA") AND ("sequencing" OR "microarray") AND ("cigarette" OR "smoke" OR "smoker" OR "tobacco" OR "smoking")

We pre-specified the number of comparisons and the following study design priorities in descending order: **(i)** intrauterine smoke exposure in any tissue/source from human offspring (maternal measurements not prioritized), **(ii)** intrauterine smoke exposure in lung animal models, **(iii)** smoke exposure in human lung tissue from healthy subjects, **(iv)** smoke exposure in lung tissue from animal models, **(v)** human lung tissues or cell lines in disease, **(vi)** smoke exposure in any human tissue/source in healthy participants. High-throughput studies (>100 miRNAs, ideally miRNA-seq or array) and larger sample sizes were prioritized.

The titles and abstracts of over 600 papers were reviewed. We note potentially relevant miRNA profiles in the context of chronic obstructive pulmonary disease and lung cancer that were not considered, as well as highly relevant mRNA and DNA methylation studies that were not considered. Studies from our group/co-authors were not included. Review articles were not included. Multiple papers sharing the same first and corresponding author were not included to mitigate over-reporting of possible center-specific effects.

Definition of Overlap:

- For each article, we count the number of significant miRNAs reported that overlap with our fetal lung IUS-miRNAs. We consider three sets of IUS-miRNAs: the all-sample analysis

(main text **Table 2**), male samples, and female samples (sex-by-IUS interaction model results in **Supplementary File S2**).
- Because of miRNA families, a major challenge in any between-miRNA study comparison is that sequences and names may be quantified and reported differently between studies: for example, the let-7 family includes isoforms hsa-let-7a through hsa-let-7i. Alternatively, a study might report hsa-miR-146b versus hsa-miR-146b-3p versus hsa-miR-146b-5p, which may represent either miRNA precursors or different mature miRNA sequences. Under the **"Flexible miRNA Family Match"** section of each article reviewed, we also consider a less stringent search of terms by checking overlaps instead of "let-7" and "146". We report the fetal lung IUS-miRNAs matches but do not compare effect directions for this less stringent comparison.

Limitations:
1. Most papers reported only significant miRNAs instead of full summary statistics, making it difficult to assess whether there were substantial differences in the miRNA lists tested (indicative of potential major between-tissue or between-study effects) and more sensitive approaches to comparing overlaps (e.g., correlation in p-values of effect size estimates instead of a simple overlap yes/no).
2. This lack of access to full lists of miRNAs tested also makes evaluating the possible quantification/miRNA family reporting issue described in Limitation #1 more challenging.
3. In "targeted" studies (fewer than 10 miRNAs) it is not always transparent how miRNA candidates were selected.
4. Existing studies cover a broad range of populations studied, sample-size, and definitions of statistical significance; each study likely has different levels of statistical power.
5. Existing studies have a mix of sexes/genders considered. We found evidence of sex-specific IUS effects and of miRNA-outcome associations in childhood asthmatics (GACRS).
6. For comparisons to animal models, we assume name homology of miRNAs. Some databases (e.g., miRbase) are updated to maintain homology, but the sequence definitions may be not be transparent in each study.

Article 1

Vives-Usano, M.; Hernandez-Ferrer, C.; Maitre, L.; Ruiz-Arenas, C.; Andrusaityte, S.; Borràs, E.; Carracedo, Á.; Casas, M.; Chatzi, L.; Coen, M.; et al. In utero and childhood exposure to tobacco smoke and multi-layer molecular signatures in children. *BMC Med.* **2020**, *18*, 1–19.

Study Design:
- Tissue: whole blood
- Comparison: children exposed to trimester-specific maternal smoking during pregnancy and early-life household smoke exposure (n = 895)
- Number of significant miRNAs reported: 0 (FDR < 0.10)

Article 2

Herberth, G.; Bauer, M.; Gasch, M.; Hinz, D.; Röder, S.; Olek, S.; Kohajda, T.; Rolle-Kampczyk, U.; Von Bergen, M.; Sack, U.; et al. Maternal and cord blood miR-223 expression associates with prenatal tobacco smoke exposure and low regulatory T-cell numbers. *J. Allergy Clin. Immunol.* **2014**, *133*.

Study Design:
- Tissue: maternal blood and cord blood
- Comparison: maternal smoking during pregnancy (n ≈ 300 to 400)
- Number of significant miRNAs reported: 2

Identical miRNA Name Match:
- Number miRNAs detected/tested in our fetal lung dataset: 0

Flexible miRNA Family Match (Less Stringent):
- Number miRNAs family members detected/tested in our fetal lung dataset: 2
- Number significant IUS-miRNAs (all-sample analysis): 0
- Number significant IUS-miRNAs (male samples): 0
- Number significant IUS-miRNAs (female samples): 0

<u>Article 3</u>

Sekovanić, A.; Dorotić, A.; Pašalić, D.; Orct, T.; Kljaković-Gašpić, Z.; Grgec, A.S.; Stasenko, S.; Mioč, T.; Piasek, M.; Jurasović, J. The effects of maternal cigarette smoking on cadmium and lead levels, miRNA expression and biochemical parameters across the feto-placental unit. *Heliyon* **2022**, *8*.

Study Design:
- Tissue: placenta and cord blood
- Comparison: human maternal smoking during pregnancy (n = 72)
- Number of significant miRNAs reported: 5 (Table 2 of article, unadjusted $p < 0.05$)

Identical miRNA Name Match:
- Number miRNAs detected/tested in our fetal lung dataset: 0

Flexible miRNA Family Match (Less Stringent):
- Number miRNAs family members detected/tested in our fetal lung dataset: 8
- Number significant IUS-miRNAs (all-sample analysis): 0
- Number significant IUS-miRNAs (male samples): 2
  hsa-miR-146a-5p; hsa-miR-190a-5p
- Number significant IUS-miRNAs (female samples): 0

<u>Article 4</u>

Juracek, J.; Piler, P.; Janku, P.; Radova, L.; Slaby, O. Identification of microRNA signatures in umbilical cord blood associated with maternal characteristics. *PeerJ* **2019**, *2019*, 1–11.

Study Design:
- Tissue: cord blood
- Comparison: maternal smoking during pregnancy (n = 24)
- Number of significant miRNAs reported: 3 (Table 2 of article; unadjusted p-value < 0.05)

Identical miRNA Name Match:
- Number miRNAs detected/tested in our fetal lung dataset: 3
- Number significant IUS-miRNAs (all-sample analysis): 0
- Number significant IUS-miRNAs (male samples): 0
- Number significant IUS-miRNAs (female samples): 0

Flexible miRNA Family Match (Less Stringent):
- Number miRNAs family members detected/tested in our fetal lung dataset: 16
- Number significant IUS-miRNAs (all-sample analysis): 0
- Number significant IUS-miRNAs (male samples): 2
  hsa-miR-30b-5p; hsa-miR-520b-5p
- Number significant IUS-miRNAs (female samples): 0

Article 5

Dehmel, S.; Nathan, P.; Bartel, S.; El-Merhie, N.; Scherb, H.; Milger, K.; John-Schuster, G.; Yildirim, A.O.; Hylkema, M.; Irmler, M.; et al. Intrauterine smoke exposure deregulates lung function, pulmonary transcriptomes, and in particular insulin-like growth factor (IGF)-1 in a sex-specific manner. *Sci. Rep.* **2018**, *8*, 1–12.

Study Design:
- Tissue: mouse lung
- Comparison: mouse maternal cigarette exposure during pregnancy (n = 24)
- Number of significant miRNAs reported: 133
  (used top 50 provided in Supplementary Figure S3 of article, unadjusted p < 0.05)

Identical miRNA Name Match:
- Number miRNAs detected/tested in our fetal lung dataset: 7
  (assuming mmu → hsa homology)
- Number significant IUS-miRNAs (all-sample analysis): 1
  hsa-miR-543: increase with IUS in mouse, increase with IUS in human fetal lung
- Number significant IUS-miRNAs (male samples): 1
  hsa-miR-449a: increase with IUS in mouse, decrease with IUS in human fetal lung
- Number significant IUS-miRNAs (female samples): 0

Flexible miRNA Family Match (Less Stringent):

- Number miRNAs family members detected/tested in our fetal lung dataset: 57
- Number significant IUS-miRNAs (all-sample analysis): 3
  hsa-let-7e-5p; hsa-miR-34a-5p; hsa-miR-543
- Number significant IUS-miRNAs (male samples): 11
  hsa-let-7a-5p; hsa-let-7b-5p; hsa-let-7d-3p; hsa-let-7e-5p; hsa-miR-124-3p; hsa-miR-130a-5p; hsa-miR-34a-5p; hsa-miR-374c-5p; hsa-miR-449a; hsa-miR-449b-5p; hsa-miR-486-5p
- Number significant IUS-miRNAs (female samples): 0

Article 6

Willinger, C.M.; Rong, J.; Tanriverdi, K.; Courchesne, P.L.; Huan, T.; Wasserman, G.A.; Lin, H.; Dupuis, J.; Joehanes, R.; Jones, M.R.; et al. MicroRNA Signature of Cigarette Smoking and Evidence for a Putative Causal Role of MicroRNAs in Smoking-Related Inflammation and Target Organ Damage. *Circ. Cardiovasc. Genet.* **2017**, *10*, 1–12.

Study Design:
- Tissue: whole blood
- Comparison: human Current vs Former vs Never smokers (n = 1,180 to 4,805)
- Number of significant miRNAs reported: 6 (Table 2 of article, FDR < 0.10)

Identical miRNA Name Match:
- Number miRNAs detected/tested in our fetal lung dataset: 3
- Number significant IUS-miRNAs (all-sample analysis): 1
  hsa-miR-423-5p: upregulated in CvN and FvN, downregulated in CvF; upregulated with IUS
- Number significant IUS-miRNAs (male samples): 2
  hsa-miR-25-5p: upregulated in CvsN, CvsF, FvsN; upregulated in IUS
  hsa-miR-423-5p: upregulated in NvsC and NvsF, downregulated in CvsF; upregulated with IUS
- Number significant IUS-miRNAs (female samples): 0

Flexible miRNA Family Match (Less Stringent):
- Number miRNAs family members detected/tested in our fetal lung dataset: 7
- Number significant IUS-miRNAs (all-sample analysis): 1
  hsa-miR-423-5p
- Number significant IUS-miRNAs (male samples): 3
  hsa-miR-25-5p; hsa-miR-423-3p; hsa-miR-423-5p
- Number significant IUS-miRNAs (female samples): 3
  hsa-miR-1246; hsa-miR-133a-3p; hsa-miR-133b

Article 7

Wang, G.; Wang, R.; Strulovici-Barel, Y.; Salit, J.; Staudt, M.R.; Ahmed, J.; Tilley, A.E.; Yee-Levin, J.; Hollmann, C.; Harvey, B.-G.; et al. Persistence of Smoking-Induced Dysregulation of MiRNA Expression in the Small Airway Epithelium Despite Smoking Cessation. *PLoS One* **2015**, *10*, e0120824.

Study Design:
- Tissue: small airway epithelium
- Comparison: human healthy non-smokers versus healthy smokers (n = 19)
- Number of significant miRNAs reported: 34 (Table 2 of article, unadjusted p < 0.01)

Identical miRNA Name Match:
- Number miRNAs detected/tested in our fetal lung dataset: 4
- Number significant IUS-miRNAs (all-sample analysis): 1
  hsa-miR-1246: downregulated in smokers, upregulated in fetal lung with IUS
- Number significant IUS-miRNAs (male samples): 1
  hsa-miR-199a: upregulated in smokers, downregulated in male fetal lung with IUS
- Number significant IUS-miRNAs (female samples): 2
  hsa-miR-1246a: downregulated in smokers, upregulated in female fetal lung with IUS
  hsa-miR-133b: upregulated in smokers, upregulated in female fetal lung with IUS

Flexible miRNA Family Match (Less Stringent):
- Number miRNAs family members detected/tested in our fetal lung dataset: 62
- Number significant IUS-miRNAs (all-sample analysis): 3
  hsa-let-7e-5p; hsa-miR-1246; hsa-miR-675-3p
- Number significant IUS-miRNAs (male samples): 14
  hsa-let-7a-5p; hsa-let-7b-5p; hsa-let-7d-3p; hsa-let-7e-5p; hsa-miR-126-3p; hsa-miR-145-5p; hsa-miR-146a-5p; hsa-miR-193b-3p; hsa-miR-199a-3p; hsa-miR-214-3p; hsa-miR-224-5p; hsa-miR-449a; hsa-miR-449b-5p; hsa-miR-675-3p
- Number significant IUS-miRNAs (female samples): 3
  hsa-miR-1246; hsa-miR-133a-3p; hsa-miR-133b

Article 8

Schembri, F.; Sridhar, S.; Perdomo, C.; Gustafson, A.M.; Zhang, X.; Ergun, A.; Lu, J.; Liu, G.; Zhang, X.; Bowers, J.; et al. MicroRNAs as modulators of smoking-induced gene expression changes in human airway epithelium. *Proc. Natl. Acad. Sci. U. S. A.* **2009**, *106*, 2319–2324.

Study Design:
- Tissue: bronchial epithelium
- Comparison: human smoker versus non-smoker (n = 20)
- Number of significant miRNAs reported: 28 (Figure 1 of article, unadjusted p < 0.05)

Identical miRNA Name Match:
- Number miRNAs detected/tested in our fetal lung dataset: 2

- Number significant IUS-miRNAs (all-sample analysis): 0
- Number significant IUS-miRNAs (male samples): 0
- Number significant IUS-miRNAs (female samples): 0

Flexible miRNA Family Match (Less Stringent):
- Number miRNAs family members detected/tested in our fetal lung dataset: 54
- Number significant IUS-miRNAs (all-sample analysis): 5
  hsa-let-7e-5p; hsa-miR-10b-5p; hsa-miR-125b-5p; hsa-miR-140-3p; hsa-miR-34a-5p
- Number significant IUS-miRNAs (male samples): 14
  hsa-let-7a-5p; hsa-let-7b-5p; hsa-let-7d-3p; hsa-let-7e-5p; hsa-miR-124-3p; hsa-miR-125a-3p; hsa-miR-125a-5p; hsa-miR-125b-5p; hsa-miR-140-3p; hsa-miR-145-5p; hsa-miR-146a-5p; hsa-miR-26b-3p; hsa-miR-30b-5p; hsa-miR-34a-5p
- Number significant IUS-miRNAs (female samples): 1
  hsa-miR-10b-5p

Article 9

Huang, J.; Jiang, W.; Tong, X.; Zhang, L.; Zhang, Y.; Fan, H.; Ding, J. Identification of gene and microRNA changes in response to smoking in human airway epithelium by bioinformatics analyses. *Med. (United States)* **2019**, *98*.

Study Design:
- Tissue: bronchial epithelium
- Comparison: human smoker versus non-smoker (n = 22)
- Number of significant miRNAs reported: 6 (Table 3; hub genes with between-group top 10 |logFC|s and unadjusted p-value < 0.05)

Identical miRNA Name Match:
- Number miRNAs detected/tested in our fetal lung dataset: 6
- Number significant IUS-miRNAs (all-sample analysis): 0
- Number significant IUS-miRNAs (male samples): 0
- Number significant IUS-miRNAs (female samples): 0

Flexible miRNA Family Match (Less Stringent):
- Number miRNAs family members detected/tested in our fetal lung dataset: 14
- Number significant IUS-miRNAs (all-sample analysis): 0
- Number significant IUS-miRNAs (male samples): 3
  hsa-miR-106b-5p; hsa-miR-500a-5p; hsa-miR-500b-5p
- Number significant IUS-miRNAs (female samples): 0

Article 10

Izzotti, A.; Calin, G.A.; Arrigo, P.; Steele, V.E.; Croce, C.M.; De Flora, S. Downregulation of microRNA expression in the lungs of rats exposed to cigarette smoke. *FASEB J.* **2009**, *23*, 806–812.

Study Design:
- Tissue: rat lung
- Comparison: cigarette exposure (n = 16)
- Number of significant miRNAs reported: 25 (Table 1 of downregulated with >3-fold and single upregulated miRNA, unadjusted $p < 0.05$)

Identical miRNA Name Match:
- Number miRNAs detected/tested in our fetal lung dataset: 0
  (assuming rno → hsa homology)

Flexible miRNA Family Match (Less Stringent):
- Number miRNAs family members detected/tested in our fetal lung dataset: 54
- Number significant IUS-miRNAs (all-sample analysis): 5
  hsa-let-7e-5p; hsa-miR-10b-5p; hsa-miR-125b-5p; hsa-miR-140-3p; hsa-miR-34a-5p
- Number significant IUS-miRNAs (male samples): 14
  hsa-let-7a-5p; hsa-let-7b-5p; hsa-let-7d-3p; hsa-let-7e-5p; hsa-miR-124-3p; hsa-miR-125a-3p; hsa-miR-125a-5p; hsa-miR-125b-5p; hsa-miR-140-3p; hsa-miR-145-5p; hsa-miR-146a-5p; hsa-miR-26b-3p; hsa-miR-30b-5p; hsa-miR-34a-5p
- Number significant IUS-miRNAs (female samples): 1
  hsa-miR-10b-5p

Supplementary File S2: Sex-by-Interaction Results

About: We evaluated potential sex-specific associations between IUS and miRNA profiles by allowing for a sex-by-IUS interaction term to our regression model (DESeq2). The following tables respectively show statistically significant interactions (**Table S1**, null hypothesis H_0: interaction effect coefficient = 0), miRNAs with significant IUS-effects among males (**Table S2**, H_0: male IUS effect = 0), and miRNAs with significant IUS-effects among females (**Table S3**, H_0: female IUS effect = 0). Each table is sorted by effect size.

Example of interpreting effects: Due to the way we specified sex in the model, the estimated average [effect of IUS-exposure in male samples] = [IUS Effect in female samples] + [sex-by-IUS interaction effect], with positive log_2FC indicating increases in log(mean miRNA levels) after accounting for other covariates.

Thus, a $log_2FC > 0$ interaction effect below indicates that the mean IUS effect in males is "more positive" than that for females, whereas a $log_2FC < 0$ interaction effect indicates that the IUS effect is "more negative". For example, hsa-miR-1246 is significantly differentially expressed with IUS in females ($log_2FC = 0.83$; Table S1). This miRNA has a significant, negative interaction effect (interaction $log_2FC = -0.67$; Table S3). The male effect is not significantly differentially expressed with IUS (log2FC = 0.16 not significantly different from zero; q-value = 0.40 and not in Table S2).

Table S1. Significant Sex-by-IUS Interaction Effects.

miRNA	Chrom	Interaction Effect (log2FC)	SE (log2FC)	p-value	q-value
hsa-miR-3913-5p	3	0.76	0.296	1.0e-02	0.03721
hsa-miR-3158-3p	2	0.73	0.366	4.8e-02	0.08872
hsa-miR-1247-5p	6	0.58	0.212	6.5e-03	0.03102
hsa-miR-6507-5p	2	0.55	0.238	2.1e-02	0.05465
hsa-miR-195-3p	8	0.48	0.201	1.8e-02	0.05230
hsa-miR-363-5p	X	0.47	0.242	5.2e-02	0.09607
hsa-miR-331-5p	4	0.37	0.165	2.7e-02	0.06272
hsa-miR-3605-3p	1	0.35	0.139	1.1e-02	0.03961
hsa-miR-1323	11	0.32	0.134	1.7e-02	0.05062
hsa-miR-375-3p	13	0.30	0.119	1.1e-02	0.03912
hsa-miR-423-5p	8	0.30	0.093	1.5e-03	0.01273
hsa-miR-424-3p	X	0.29	0.099	2.8e-03	0.01945
hsa-miR-1306-5p	14	0.29	0.147	4.7e-02	0.08872

hsa-miR-27b-3p	22	0.29	0.075	1.2e-04	0.00343
hsa-let-7d-3p	21	0.27	0.093	3.6e-03	0.02233
hsa-miR-654-5p	6	0.27	0.124	3.0e-02	0.06591
hsa-let-7a-5p	15,21,3	0.27	0.106	1.2e-02	0.04039
hsa-miR-200b-5p	1	0.26	0.083	1.6e-03	0.01312
hsa-miR-423-3p	8	0.26	0.091	3.9e-03	0.02315
hsa-miR-193a-5p	9	0.25	0.112	2.6e-02	0.06272
hsa-miR-126-3p	22	0.25	0.077	1.2e-03	0.01181
hsa-miR-224-5p	X	0.25	0.108	2.2e-02	0.05465
hsa-miR-1301-3p	12	0.24	0.102	1.8e-02	0.05300
hsa-miR-200c-3p	3	0.23	0.088	9.0e-03	0.03434
hsa-miR-340-3p	17	0.23	0.091	1.3e-02	0.04310
hsa-miR-200b-3p	1	0.21	0.062	5.8e-04	0.00729
hsa-miR-652-3p	X	0.21	0.081	1.1e-02	0.03912
hsa-miR-485-5p	6	0.20	0.099	4.7e-02	0.08872
hsa-miR-125a-5p	11	0.16	0.074	2.9e-02	0.06495
hsa-miR-151a-3p	20	0.16	0.078	4.5e-02	0.08771
hsa-miR-15b-5p	16	-0.20	0.074	8.2e-03	0.03295
hsa-miR-323a-3p	5	-0.21	0.100	3.5e-02	0.07371
hsa-miR-152-3p	9	-0.22	0.095	2.1e-02	0.05465
hsa-miR-199a-3p	1,10	-0.22	0.059	1.8e-04	0.00455
hsa-miR-382-3p	6	-0.26	0.135	5.2e-02	0.09561
hsa-miR-376c-3p	6	-0.26	0.100	8.1e-03	0.03295
hsa-miR-654-3p	6	-0.27	0.102	8.8e-03	0.03432
hsa-miR-136-3p	5	-0.28	0.125	2.7e-02	0.06272
hsa-miR-20b-5p	X	-0.28	0.134	3.8e-02	0.07650
hsa-miR-20a-5p	5	-0.28	0.118	1.7e-02	0.05062
hsa-miR-495-3p	6	-0.30	0.136	2.8e-02	0.06372
hsa-miR-574-3p	16	-0.30	0.144	3.7e-02	0.07650
hsa-miR-19b-3p	5,X	-0.30	0.115	8.0e-03	0.03295
hsa-miR-337-3p	5	-0.31	0.138	2.7e-02	0.06272

hsa-miR-15b-3p	16	-0.31	0.140	2.8e-02	0.06484
hsa-miR-324-5p	8	-0.31	0.127	1.5e-02	0.04607
hsa-miR-598-3p	19	-0.31	0.150	3.8e-02	0.07650
hsa-miR-18a-5p	5	-0.32	0.160	4.7e-02	0.08872
hsa-miR-369-3p	6	-0.32	0.101	1.3e-03	0.01258
hsa-miR-30b-5p	20	-0.32	0.103	1.7e-03	0.01324
hsa-miR-328-3p	8	-0.34	0.116	3.6e-03	0.02233
hsa-miR-378c	2	-0.34	0.146	2.0e-02	0.05417
hsa-miR-127-3p	5	-0.34	0.147	2.0e-02	0.05417
hsa-miR-582-5p	17	-0.35	0.167	3.9e-02	0.07652
hsa-miR-181c-5p	10	-0.35	0.139	1.2e-02	0.04085
hsa-miR-532-5p	X	-0.35	0.115	2.3e-03	0.01632
hsa-miR-675-5p	2	-0.36	0.172	3.8e-02	0.07652
hsa-miR-1295a	1	-0.36	0.165	3.0e-02	0.06591
hsa-miR-127-5p	5	-0.36	0.157	2.0e-02	0.05417
hsa-miR-339-5p	19	-0.37	0.132	5.5e-03	0.02781
hsa-miR-181d-5p	11	-0.37	0.130	4.1e-03	0.02384
hsa-miR-378a-3p	17	-0.38	0.146	1.0e-02	0.03720
hsa-miR-1271-5p	17	-0.38	0.152	1.2e-02	0.04039
hsa-let-7e-3p	11	-0.38	0.137	5.1e-03	0.02722
hsa-miR-409-5p	6	-0.39	0.159	1.3e-02	0.04310
hsa-miR-4286	19	-0.40	0.174	2.3e-02	0.05827
hsa-miR-19a-3p	5	-0.42	0.162	8.9e-03	0.03432
hsa-miR-889-3p	6	-0.43	0.162	7.7e-03	0.03295
hsa-miR-455-5p	22	-0.43	0.118	2.7e-04	0.00600
hsa-miR-34a-5p	1	-0.44	0.122	3.3e-04	0.00626
hsa-miR-365a-3p	7	-0.45	0.165	6.7e-03	0.03102
hsa-miR-93-3p	19	-0.45	0.153	3.2e-03	0.02154
hsa-miR-410-3p	6	-0.46	0.201	2.2e-02	0.05465
hsa-miR-487a-3p	6	-0.46	0.198	1.9e-02	0.05300
hsa-miR-500a-5p	X	-0.47	0.172	6.0e-03	0.02955

hsa-miR-887-3p	17	-0.47	0.227	3.6e-02	0.07650
hsa-miR-223-3p	X	-0.48	0.145	8.4e-04	0.00938
hsa-miR-106b-5p	19	-0.48	0.097	6.4e-07	0.00013
hsa-miR-500b-5p	X	-0.49	0.172	4.4e-03	0.02452
hsa-miR-504-5p	X	-0.50	0.186	7.2e-03	0.03240
hsa-miR-296-5p	14	-0.51	0.238	3.2e-02	0.06952
hsa-miR-641	11	-0.52	0.252	3.9e-02	0.07652
hsa-miR-708-5p	3	-0.53	0.134	7.3e-05	0.00343
hsa-miR-135b-3p	1	-0.55	0.257	3.4e-02	0.07309
hsa-miR-1287-5p	2	-0.56	0.206	6.8e-03	0.03102
hsa-miR-1296-5p	2	-0.57	0.201	4.6e-03	0.02530
hsa-miR-656-3p	6	-0.58	0.168	5.8e-04	0.00729
hsa-miR-299-3p	5	-0.58	0.209	5.4e-03	0.02775
hsa-miR-532-3p	X	-0.59	0.154	1.2e-04	0.00343
hsa-miR-380-3p	5	-0.60	0.178	7.2e-04	0.00850
hsa-miR-539-5p	6	-0.60	0.198	2.3e-03	0.01632
hsa-miR-331-3p	4	-0.64	0.145	1.2e-05	0.00120
hsa-miR-551b-3p	16	-0.66	0.248	7.5e-03	0.03290
hsa-miR-1246	12	-0.67	0.291	2.0e-02	0.05418
hsa-miR-675-3p	2	-0.74	0.358	3.9e-02	0.07652
hsa-miR-585-3p	17	-0.76	0.357	3.4e-02	0.07309
hsa-miR-744-3p	8	-0.76	0.322	1.8e-02	0.05300
hsa-miR-520b-5p	11	-0.77	0.345	2.5e-02	0.06150
hsa-miR-491-5p	21	-0.80	0.342	1.9e-02	0.05300
hsa-miR-133a-3p	10,14	-0.87	0.223	9.3e-05	0.00343
hsa-miR-3613-3p	4	-0.89	0.365	1.5e-02	0.04582
hsa-miR-133b	18	-0.91	0.224	4.7e-05	0.00317
hsa-miR-1248	16	-0.92	0.259	3.7e-04	0.00626
hsa-miR-874-5p	17	-0.92	0.294	1.7e-03	0.01324
hsa-miR-12136	1	-0.94	0.288	1.1e-03	0.01157
hsa-miR-193b-3p	7	-0.96	0.329	3.7e-03	0.02233

hsa-miR-1283	11	-0.99	0.284	5.1e-04	0.00729
hsa-miR-154-5p	6	-1.01	0.283	3.7e-04	0.00626
hsa-miR-130a-5p	2	-1.02	0.319	1.4e-03	0.01273
hsa-miR-518e-3p	11	-1.22	0.352	5.4e-04	0.00729

<u>Table S2.</u> Significant IUS-exposure in male samples, based on model allowing for sex-by-IUS interaction. Empirical miRNA-microarray Spearman correlations are within male samples only and the + or - following each gene symbol indicates direction of correlation.

Higher Levels In	miRNA	Chrom	IUS Effect (log2FC)	SE (log2FC)	p-value	q-value	Top Microarray Correlations
IUS	hsa-miR-3158-3p	2	0.6	0.24	0.012	0.063	STAU1+; PCNX1+; NCBP2+; ARMC10+; ADH1C+; BUD23+; RND3+; EMP3-
	hsa-miR-372-3p	11	0.5	0.21	0.02	0.077	CCN4+; RASSF3+; CNR2+; FAM174B+; CLEC11A+; CYP4F2+; OR1G1+; ACTN3-
	hsa-miR-3913-5p	3	0.49	0.2	0.012	0.063	ZNF16+; ZNF518B+; CASR+; HFM1+; MCCC2+; MCCC2+; GABPB1+; LYN+
	hsa-miR-6507-5p	2	0.43	0.16	0.0065	0.046	FAM229B+; ME2-; MDM4-; GTDC1-; IDH3A-; ABHD4+; BMF+; SMCHD1-
	hsa-miR-1323	11	0.43	0.09	1.8e-06	0.00017	IL31RA+; MRPS22+; PTTG1+; TTK+; CCNB2+; MDH1+; FBXO5+; GGH+
	hsa-miR-146a-5p	17	0.39	0.15	0.012	0.063	CFAP221+; ABCA3+; SLC4A4+; CRYAB-; STAT3+; GCH1+; ETV6+; CHPF-
	hsa-miR-1247-5p	6	0.36	0.14	0.01	0.06	LOX-; OGN-; FNDC1-; PPM1K-; NKAIN4+; NAV3-; SGCE-; APOL6+
	hsa-miR-331-5p	4	0.31	0.11	0.0053	0.043	ARL4C+; ATXN7L2+; INSYN2A-; DRD2+; GNAZ+; FMO1-; OXLD1+; HUS1B+
	hsa-miR-6788-3p	9	0.29	0.12	0.012	0.063	RIPK2+; MAP3K8+; SLITRK1-; NLGN4X-; PPP2R2B-; C2CD5-; ACSL4+; PCDH9+
	hsa-miR-423-5p	8	0.29	0.062	3.1e-06	0.00018	CPA1-; VEGFB+; ZNF672+; FAM92A+; MYL3+; TMEM53+; CKAP4+; LANCL1-
	hsa-miR-3613-5p	4	0.28	0.088	0.0013	0.017	HIST1H3J+; SREK1IP1-; BBS12-; HIST1H2BI+; BRWD3-; HIST1H2AE+; COX20-; MOSPD2-
	hsa-miR-370-3p	5	0.28	0.12	0.022	0.079	SP5-; DNAH14+; TTC23-; CIR1-; TBX2-; RALGAPB+; GDF5OS-; METTL21A+
	hsa-miR-431-5p	5	0.24	0.11	0.025	0.083	CXCL16+; TTLL1-; SP6+; APOF-; ATP8A2-; SLIT1-; MYO1D+; FGF12-
	hsa-miR-424-3p	X	0.24	0.066	0.00027	0.006	NCAN-; TAS1R2-; NPC1L1-; FOXG1-; ATN1+; DCAF12L1-; MUC17-; TMEM201+
	hsa-miR-224-5p	X	0.23	0.072	0.0014	0.017	GABRE+; REEP2+; AKT1S1+; ADRA1D+; PRELP+; SMIM5-; DGKK-; PLEK2-
	hsa-miR-25-5p	19	0.23	0.08	0.004	0.035	GMNN+; CNTN3-; UFD1+; GAREM2+; ORC1+; MBNL3+; CCNE2+; PCNA+
	hsa-miR-3605-3p	1	0.23	0.092	0.013	0.065	FAM111B-; EEF1AKMT3+; CHTOP-; NSUN7+; MAP3K7CL+; HSPB11-; SLC10A7+; SPAG8+
	hsa-miR-2110	2	0.23	0.082	0.0057	0.045	DDX23+; SP3+; LEXM+; TEX13A+; CIB1+; RBM43-; CLEC17A-; RGS12+
	hsa-miR-24-3p	10,22	0.21	0.085	0.014	0.067	PRKD1-; TSC22D1-; ORAI1+; DNAJC2+; LACTB+; CDC42EP1+; IFT57+; PPP1R14A+
	hsa-miR-125a-3p	11	0.21	0.068	0.0022	0.024	NFATC3+; SEPTIN1+; EFCAB11+; UNC5B+; ARRB2+; PAX3-; WDR61+; KCNB2+

	hsa-let-7e-5p	11	0.21	0.059	0.00044	0.0081	TSPAN11+; SEC23A+; CLEC11A+; HYI+; SLC45A4+; RNASE3-; RFX6-; SLC29A3-
	hsa-miR-320d	4,X	0.19	0.087	0.031	0.097	ADSS+; CIB1+; EFNB2+; SPACA1+; NAIF1+; AQP1+; FCAR-; LCN8-
	hsa-let-7b-5p	15	0.18	0.085	0.03	0.092	HYI+; TMEM231+; CEP57L1+; CFAP36+; HK2-; BTBD1-; PEBP4+; DNAJC19+
	hsa-miR-1301-3p	12	0.18	0.068	0.0074	0.049	SULT2B1-; FSD1L+; SEC23A-; OR1K1-; GPHA2-; PTK2B+; HSP90AB1-; KRTAP19-4+
	hsa-let-7a-5p	15,21,3	0.18	0.071	0.012	0.063	SLC29A3-; ANKRD13C+; CLEC11A+; EXOC1+; FAM193B-; MRPS10+; IL1RL2-; PEBP4+
	hsa-miR-574-5p	16	0.18	0.068	0.01	0.06	PKP1-; CEP85L-; SYCP2-; TMEM38B-; OTP-; SEPTIN10-; PDCD2L-; RNPEP+
	hsa-let-7d-3p	21	0.17	0.062	0.0073	0.049	NRIP3+; GDPD4+; HDAC2-; RABGAP1-; RSPH6A-; GPSM2-; B3GALT5-; OR10H3+
	hsa-miR-485-5p	6	0.16	0.066	0.017	0.073	ZNF3+; KDM3A-; MYH10+; SH3BP5L-; GPR157+; ZNF181+; RANBP9-; CCP110-
	hsa-miR-27b-3p	22	0.15	0.05	0.0023	0.024	CALN1-; ZNF491-; ERO1A+; ACO2+; SNX33-; JMJD8+; NKIRAS2+; PUM1-
	hsa-miR-126-3p	22	0.14	0.051	0.0046	0.038	LACTB+; TNFRSF21+; CPA4-; ABAT-; BMP1-; CX3CL1+; TRMT6+; SELENOI+
	hsa-miR-200b-5p	1	0.14	0.055	0.012	0.063	KRTAP19-4+; OR2AP1+; IL5+; ANKRD33-; LRRC70+; DNALI1+; ABHD2+; MMP16-
	hsa-miR-423-3p	8	0.13	0.061	0.027	0.088	XPR1+; RALBP1-; MYOZ3-; ZNF473+; HMCN2-; CCDC182+; SERPINB7+; CA3-
	hsa-miR-652-3p	X	0.12	0.054	0.023	0.079	LRRC32+; LANCL2-; VPS37A+; SLC30A1-; NSL1-; ARAF+; P4HA1+; PTRH1-
	hsa-miR-200b-3p	1	0.12	0.041	0.0035	0.035	IRF8+; HMG20B+; NFS1+; QARS+; PIN1+; EIF3G+; USF1+; STARD3+
	hsa-miR-125a-5p	11	0.12	0.05	0.02	0.077	NDUFAF4+; LACTB+; TRAF3+; GZMH-; GNL3+; NECTIN1+; RPS23+; GPC5+
non-exposed	hsa-miR-101-3p	1,20	-0.14	0.059	0.022	0.079	IGSF1+; PPP4C-; DAZAP1-; UTF1-; NDUFAF6-; NFATC1-; CKAP4-; SMNDC1-
	hsa-miR-199a-3p	1,10	-0.14	0.039	0.00025	0.006	CA3+; IL1F10+; GNPDA2+; SEC23B-; NMD3+; GPR3+; ATF6-; PBRM1-
	hsa-miR-374c-5p	X	-0.15	0.065	0.021	0.077	MFSD6-; TLR5-; RALBP1+; DUOX1-; BCLAF3-; KIF20B+; HAO2+; RPL36+
	hsa-miR-369-3p	6	-0.16	0.067	0.02	0.077	USP6NL-; FAM110A-; SCN1A-; IL1RAP-; GNAQ-; HHLA1+; MTRNR2L11+; DNAJB9-
	hsa-miR-484	8	-0.17	0.071	0.015	0.068	KIR2DL4+; KIR2DL4+; MEPE+; CLDN34-; CHRNA4-; NXPE2+; GVQW3+; TOR3A+
	hsa-miR-328-3p	8	-0.18	0.077	0.021	0.077	SEPTIN10-; C1ORF210+; SLC6A2-; NSRP1-; NEXN-; IL23A+; CEACAM20-; YIPF2+
	hsa-miR-30b-5p	20	-0.19	0.069	0.0067	0.047	NR1D1+; SLC22A1+; ZNF699+; PEX2+; ATP6V1D+; TBCCD1-; ZNF347+; GMDS-
	hsa-miR-20a-5p	5	-0.19	0.079	0.015	0.068	ISCU+; LST1+; LST1+; LST1+; PIK3R5+; C1QL2+; DNAJB5-; TTK+
	hsa-miR-145-5p	17	-0.19	0.073	0.0081	0.052	KRT80-; SFR1+; MOGAT1+; ME1+; BRSK1-; SCN1A+; PRKRIP1-; ADRB2+
	hsa-miR-152-3p	9	-0.21	0.064	9e-04	0.013	C15ORF39-; PCDH8+; PRKD3-; ENTPD1+; MBNL3-; LPA+; SKA3-; TMSB15A-
	hsa-miR-214-3p	1	-0.21	0.097	0.029	0.09	PLK2-; SARM1+; HDAC1-; CYP2C9+; DUOX1-; DUSP21+; KDM1A-; RECQL+
	hsa-miR-378c	2	-0.23	0.097	0.021	0.077	TSG101-; PYDC2+; POU2AF1+; OR1G1-; ZMAT4-; WASHC3+; FAM161A-; SETDB2-
	hsa-miR-1271-5p	17	-0.23	0.1	0.021	0.077	VAPB-; CLEC2B+; FAM92A-; ZNF554-; DPH2+; LAGE3-; MAST1-; LPGAT1-
	hsa-miR-598-3p	19	-0.24	0.1	0.019	0.077	PC+; STOX1+; PIM1-; STIM2-; REEP1+; FTSJ3+; GPRASP1+; SYN2+
	hsa-miR-125b-5p	14,3	-0.24	0.071	0.00063	0.0093	TTK-; TFDP2-; CCNB1-; MIPOL1+; CDCA7L-; NCAPD2-; HIST1H1B-; BUB1B-

hsa-miR-455-5p	22	-0.24	0.079	0.002	0.023	FMN2-; AGO1-; CCAR2-; UBTF-; SKI-; VTN+; SNX1-; CABLES2-
hsa-miR-656-3p	6	-0.26	0.11	0.021	0.077	RALYL-; DSCAML1+; BEGAIN+; OTOF+; REG1B-; QRFP+; EXD3+; RASSF1+
hsa-miR-135a-5p	15,4	-0.26	0.11	0.016	0.072	ANAPC16-; ZSCAN30+; HERC1-; THADA-; SLC25A16-; TMEM119-; MBOAT2-; BPHL-
hsa-miR-378a-3p	17	-0.27	0.097	0.0062	0.046	TSG101-; POU2AF1+; SETDB2-; WASHC3+; FAM161A-; PYDC2+; CPA1+; YAE1+
hsa-miR-500a-5p	X	-0.28	0.12	0.016	0.072	SEL1L2+; GAREM2-; SLC35E1-; THOC1-; FRMD3+; USP30-; AQP5-; HHLA1-
hsa-miR-500b-5p	X	-0.28	0.11	0.013	0.065	SEL1L2+; GAREM2-; USP30-; SLC35E1-; FRMD3+; THOC1-; CABP7-; PCSK4-
hsa-miR-708-5p	3	-0.28	0.089	0.0015	0.018	CALB1+; APLNR-; LIAS+; FIZ1-; FANCE-; PLEKHA8-; CPNE4+; CYP3A5+
hsa-miR-19a-3p	5	-0.3	0.11	0.0062	0.046	SERPINA11+; AQP8+; HEG1-; EPB41L1-; DNAH10-; NR2C2AP+; TEX2-; MAGEB6+
hsa-miR-26b-3p	13	-0.31	0.11	0.0036	0.035	ACER3+; FAM24A+; DNAJB9+; FAM83B+; HOXA6+; RHPN2+; LAPTM5-; RMND1+
hsa-miR-324-5p	8	-0.31	0.085	0.00023	0.006	ARSF-; KRBOX4+; RNF114-; RMND1+; PIEZO2-; UBTD2-; DYNC2LI1+; VAMP5-
hsa-miR-106b-5p	19	-0.32	0.065	7.2e-07	0.00017	ALB+; AHSG+; APOA2+; AFP+; CGA+; CYP19A1+; DHX8-; APOB+
hsa-miR-532-5p	X	-0.33	0.077	2.2e-05	0.00094	TTN+; ELOVL5-; EIF4G1-; TLCD3A-; PMEL+; INSIG1-; ACMSD+; CLEC12A+
hsa-miR-140-3p	8	-0.33	0.15	0.025	0.084	HACE1+; IL1R1-; ENTPD7-; GAP43+; PTPRM+; RAP2C-; TMTC3-; S100B+
hsa-miR-190a-5p	7	-0.33	0.14	0.021	0.077	ASPA+; PIK3R5+; EMILIN1-; FRYL-; GDPD5-; HES7+; PKN3+; PRSS3+
hsa-miR-4286	19	-0.33	0.12	0.0042	0.036	AZGP1+; TAF5+; NELFCD+; RFT1+; SLC22A1+; IL17F+; ITGAV+; ZIK1+
hsa-miR-1287-5p	2	-0.33	0.14	0.015	0.068	CYP4F12+; EYS-; SCNM1-; ADAM21-; ANKRD13B+; TGFBR2-; DUSP10+; DHX36+
hsa-miR-664a-3p	1	-0.33	0.13	0.011	0.063	ALB-; FXYD6-; ARHGEF9-; MLANA+; SEL1L3+; CENPO-; UBXN2A+; ABCA4+
hsa-miR-380-3p	5	-0.34	0.12	0.0039	0.035	CCP110-; PARP11-; PLK4-; PRPS2-; FILIP1+; SLC26A6-; HIST1H1D-; HIST1H4F-
hsa-miR-19b-3p	5,X	-0.35	0.076	6.3e-06	0.00031	LAMB4+; STARD9-; NAT8+; TREH+; LCE2D+; COL12A1-; CGA+; CCDC146-
hsa-miR-1296-5p	2	-0.35	0.13	0.0092	0.056	TUBGCP2+; SND1+; RNF213+; FCHSD1+; SOX18-; PISD+; THPO-; RAE1+
hsa-miR-532-3p	X	-0.35	0.1	0.00064	0.0093	ANKRD2+; GLRA1-; HES3+; GGH-; PRRC1-; NR2F1-; PIEZO2-; OVOL2+
hsa-miR-4284	19	-0.36	0.15	0.018	0.076	POU4F1-; ABCC8+; TAF11-; FBXO27+; SLC37A1-; POLR2G-; SIGLEC10+; EFHD1+
hsa-miR-93-3p	19	-0.36	0.1	0.00042	0.0081	SOWAHD+; FAM170B+; HCN2+; APOC3+; APOC3+; TH+; INSC+; DUSP27+
hsa-miR-365a-3p	7	-0.38	0.11	0.00059	0.0093	DHODH+; RFX7-; MXRA7+; M6PR+; SCUBE1-; MASTL-; EP300-; RELL1+
hsa-miR-34a-5p	1	-0.38	0.081	2.4e-06	0.00017	KIFC3-; CDH7-; JMY+; TMEM211+; SCGB1D4+; SLC46A2+; MXI1+; RAB8B+
hsa-miR-1248	16	-0.39	0.17	0.026	0.085	EPHX2-; TICRR+; NR3C2+; RAB27A+; WDR76+; WNT5A-; OR2G2+; DUOXA1+
hsa-miR-451a	8	-0.4	0.16	0.014	0.067	HBD+; C14ORF28+; CPEB4+; LHX8+; NR4A3+; AGFG2+; CERT1+; BEST1+
hsa-miR-1283	11	-0.44	0.19	0.019	0.077	TEX43+; CLEC4E+; ADAM7+; TMEM207+; ARSK+; CAPN15-; CTBP2-; FGF4+
hsa-miR-331-3p	4	-0.47	0.097	1.7e-06	0.00017	MED24-; FGD5-; ADAMTS10-; IP6K1-; ST8SIA5+; CLIP3-; FAM170B+; KIZ-
hsa-miR-551b-3p	16	-0.48	0.17	0.0039	0.035	EFNA1-; SLC10A5+; HLCS-; KLK11-; LANCL1-; CAST-; ZNF529+; SPR-
hsa-miR-486-5p	19	-0.49	0.18	0.0058	0.045	ALAS2+; SLC25A37+; HBD+; DCAF12+; RUNDC3A+; HEMGN+; TENT5C+; BPGM+

hsa-miR-142-3p	9	-0.5	0.23	0.025	0.084	TMEM51+; PHLDB2-; FPR2+; B3GNT5+; ANPEP-; TENM3-; AMOT-; FRY-
hsa-miR-520b-5p	11	-0.52	0.23	0.023	0.079	STK17B+; TREH+; LEF1+; XIAP-; TMEM100-; VIPAS39+; SNRPA+; CDCA7+
hsa-miR-518e-3p	11	-0.55	0.24	0.021	0.077	PRR30+; QDPR-; SLC22A25-; ZAP70+; C10ORF55+; ICOS+; NRG3-; RAG1+
hsa-miR-449a	17	-0.57	0.26	0.029	0.09	C20ORF85+; FAM81A+; SMC2-; AZIN2+; C1ORF158+; TMEM212+; CDC20B+; CFAP54+
hsa-miR-449b-5p	17	-0.58	0.26	0.028	0.09	C20ORF85+; SMC2-; AZIN2+; C1ORF158+; FAM81A+; TMEM212+; CDC20B+; CFAP54+
hsa-miR-766-3p	X	-0.6	0.23	0.0083	0.053	GTSF1L+; ZW10-; ZFAND3-; AC079594.2+; PACC1-; MC3R-; CLRN1+; ABCA13-
hsa-miR-29a-3p	19	-0.61	0.15	6.5e-05	0.0024	ME1+; PCSK4-; C3ORF49+; TM9SF3+; TAOK3-; OR52N1+; CASTOR2-; CASTOR2-
hsa-miR-29c-3p	1	-0.62	0.21	0.0035	0.035	PTGR1-; KCNIP3+; TPM3+; IL2RB+; LPGAT1+; PAEP+; CDH16-; GNLY+
hsa-miR-124-3p	14,19	-0.7	0.27	0.0088	0.055	CNTD1+; RBFOX1+; SERPINI2+; BTG2+; CENPL-; SERPINB5+; GAP43+; FMN2+
hsa-miR-154-5p	6	-0.7	0.19	0.00022	0.006	ZBP1+; CPLANE1-; UGT8+; MAP3K21+; CERS4-; KIAA1257-; ORMDL2-; ZYG11A+
hsa-miR-130a-5p	2	-0.74	0.21	0.00049	0.0084	ACSS3+; CIR1+; RANBP9+; ADIG+; MCRS1-; SLC28A1+; CLDN15+; SCGB1A1+
hsa-miR-193b-3p	7	-0.82	0.22	2e-04	0.006	TREML1-; HDDC3+; HADH-; LUC7L+; GTSF1L+; GUCA1B+; DLK2-; CCDC73+
hsa-miR-196b-5p	19	-0.82	0.35	0.019	0.077	UBL5+; CRTAC1+; CD5-; UTS2R-; OR14C36+; TAS2R16+; ZXDC-; GRK3-
hsa-miR-675-3p	2	-0.87	0.24	0.00029	0.0061	BLVRB-; PRR23B+; CCDC178-; RNF20-; ZNF415-; ADAMTS17+; CDCA8-; SPATA1-

Table S3. Significant IUS-exposure in female samples, based on model allowing for sex-by-IUS interaction. Empirical miRNA-microarray Spearman correlations are within female samples only. Empirical miRNA-microarray Spearman correlations are within male samples only and the + or - following each gene symbol indicates direction of correlation.

Higher Levels In	miRNA	Chrom	IUS Effect (log2FC)	SE (log2FC)	p-value	q-value	Top Microarray Correlations
IUS	hsa-miR-1246	12	0.83	0.22	0.00012	0.046	USP34-; SLC26A4+; PROS1+; SLC32A1-; SLC30A5-; ACSL1-; IRAK4+; CCL19+
	hsa-miR-133a-3p	10,14	0.61	0.17	0.00027	0.046	TRADD+; IL33+; ZSCAN32+; ZNF574-; CREBBP-; POU5F2+; SLC6A16-; ARHGEF11-
	hsa-miR-133b	18	0.6	0.17	0.00032	0.046	TRADD+; IL33+; ZSCAN32+; POU5F2+; ZNF574-; TSPO+; ARHGEF11-; ZC3H12D+
non-exposed	hsa-miR-10b-5p	12	-0.54	0.16	0.00059	0.063	POLR3K+; DFFB+; MYORG-; YEATS4-; STAB2+; CLPTM1-; SMPD4-; NLRP5+

Supplementary File S3. DIABLO Detailed Methods/Sensitivity Analyses.

Detailed DIABLO Methods/Rationale

To augment the pairwise miRNA-mRNA comparisons (**Table S1** and **Supplementary File S1**), we also used the multiomic integration package mixOmics to integrate the residualized datasets and perform feature selection of miRNAs and mRNAs for prioritization—under the constraints that the features (i) discriminate between IUS exposed and un-exposed samples while also (ii) being correlated between the miRNA and mRNA modalities. Given the evidence of sex-specific IUS miRNA signatures, we performed this multiomic integration separately on male samples and female samples.

We input each sample's IUS exposure status, residualized, Z-transformed miRNA profiles with any nominally significant associations with IUS (q-value < 0.20), and Z-transformed mRNA profiles, into DIABLO, also known as the mixOmics multiblock partial least squares-discriminant analysis algorithm (PLS-DA; block.plsda() function). We used the "full" design matrix to prioritize miRNAs with strong mRNA connections, in contrast to miRNAs that collectively may strongly discriminate IUS exposure status but have limited empirical correlations with mRNAs (e.g., "null" design matrix). Using the mixOmics tune.block.splsda() function, model parameter selection was performed allowing 1 component, and a grid search of 5-155 mRNAs with step size of 10, and 1-10 miRNAs with a step size of 1. Ultimately, a target number of 1 miRNA and 35 mRNAs were selected for the male model, whereas 2 miRNAs and 65 mRNAs were selected for the female model. We scored model performance using the AUC, calculated with perf() function from a five-fold cross-validation with 50 repeats (AUC = 0.78 in males and 0.86 in females). We also noted the canonical correlation between modalities, displayed with plotDiablo(), to be 0.53 in the female model and 0.38 in the male model. Global miRNA-mRNA correlation may be therefore be weaker in male versus female fetal lung.

Sex-Specific Correlations

In the network figure of the main text, we show only edges with "large" Spearman correlations (**Figure 2**; $r < -0.25$ or $r > 0.40$) to minimize the visual clutter and communicate core candidate miRNA-mRNA regulatory relationships. These thresholds were derived by extracting pairwise correlations between miRNA-mRNA features selected by each DIABLO model, then taking the 10th percentile of collective negative correlation values and the 90th percentile of positive, non-zero pairwise correlations.

In contrast to the edge-pruned figures in the main text, we present full pairwise correlations in heatmaps below, represented by the color and size of the associated square. We also elected to create the correlation matrices for all four combinations of (DIABLO models) x (sample data subset) possible to demonstrate that models indeed point to possible sex-specific miRNA-mRNA regulation mechanisms (**Supplementary Figure S1**). For example, recreating the correlation matrix using the features selected from the male-only DIABLO model but with data from the female samples shows weaker correlations between features (muted colors, more white negative space in figures). Most strikingly, the miRNAs selected no longer are strongly correlated with the mRNAs. This is most apparent in the dark red columns associated with *hsa-miR-200a-3p* and

hsa-miR-29c in the female data, female model (indicative of negative correlations with mRNAs) instead becoming primarily white (no correlation) in the male data, female model figure.

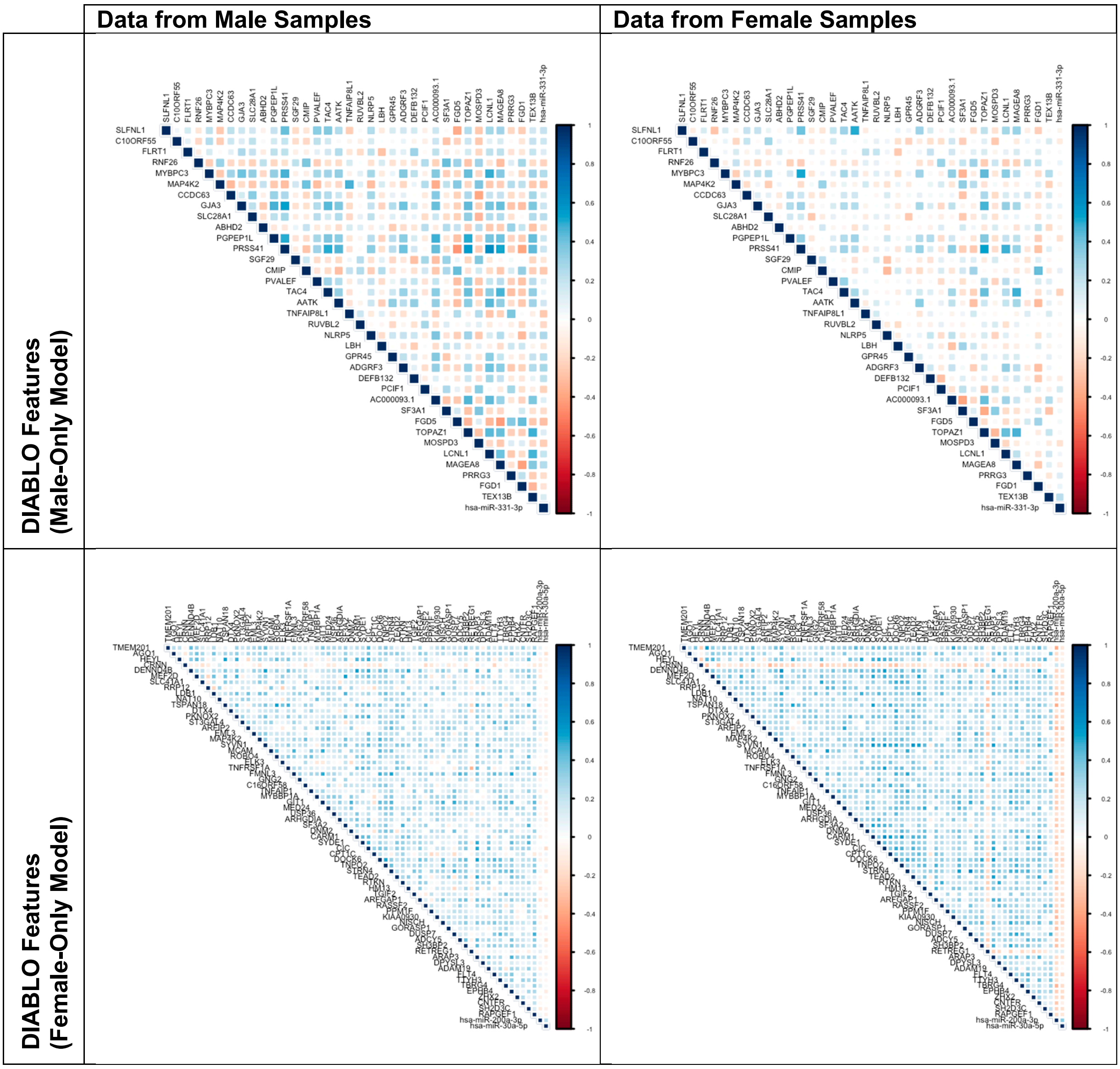

Supplementary Figure S2. Correlation heatmaps for DIABLO model miRNAs and mRNAs (rows) by sample data subset (columns).

<u>Full Gene List (Male-Only Model):</u> AATK, ABHD2, AC000093.1, ADGRF3, C10ORF55, CCDC63, CMIP, DEFB132, FGD1, FGD5, FLRT1, GJA3, GPR45, LBH, LCNL1, MAGEA8, MAP4K2, MOSPD3, MYBPC3, NLRP5, PCIF1, PGPEP1L, PRRG3, PRSS41, PVALEF, RNF26, RUVBL2, SF3A1, SGF29, SLC28A1, SLFNL1, TAC4, TEX13B, TNFAIP8L1, TOPAZ1
<u>Full Gene Lists (Female):</u> ADAM19, AGO1, ARAP3, ARFGAP1, ARFIP2, ARHGDIA, C16ORF58, CARM1, CIC, CNTFR, CPT1C, CRNN, DENND4B, DOCK6, DPYSL3, DUSP7,

ELK3, EML3, EPHB4, FLT4, FMNL3, GIT1, GNG2, GORASP1, HEYL, HM13, ITGA5, KIAA0930, L3MBTL2, LDB1, LRP10, MAP4K2, MCAM, MED24, MEF2D, MYBBP1A, NAT10, NISCH, PKNOX2, PPM1F, RAPGEF1, RASSF2, RETREG1, ROBO4, RRP12, RTKN, SF3A2, SH2D3C, SH3BP2, SLC41A1, ST3GAL4, STRN4, SYDE1, SYVN1, TBRG4, TEAD2, TGIF2, TMEM201, TNFAIP1, TNFRSF1A, TNPO2, TSPAN18, TTYH3, USP36, ZHX2

Sensitivity to DIABLO Parameters

With extensive sensitivity analyses, we reach the same conclusions as presented in the main text of our manuscript.

We performed extensive sensitivity analyses on the DIABLO results, such as changing the number of components and features selected (to the next best accuracy model suggested by tune.block.splsda), adding more components, and using the "null" design matrix (selecting miRNAs and mRNAs that discriminate on IUS status, regardless of their between-modality correlation) instead of the "full" (encourages selection of features that maximize miRNA-mRNA correlations). We present some of the main differences observed in sensitivity analyses here but reported the "full" matrix as our pre-specified goals with the analysis was to focus on miRNAs with empirical mRNA correlations and thus more evidence for miRNA regulation of gene expression.

The DIABLO output for female samples was generally robust, with the "hsa-miR-200a-3p" miRNA consistently selected in all models and similar candidate mRNA targets. Under different scenarios "hsa-miR-29c" was selected in lieu of "hsa-miR-30a."

The DIABLO output for male samples was less robust, with "hsa-miR-1323" (significantly increased with IUS in male samples) sometimes selected by DIABLO, albeit with less frequency than the "hsa-miR-331-3p" miRNA presented in the main text (significantly decreased with IUS in male samples). This lack of between-model concordance may be due to the comparatively lower canonical, overall miRNA-mRNA correlation among male samples. hsa-miR-1323 in particular had small pairwise correlations. An example network figure is below (**Supplementary Figure S2**), lowering the stringency of edge inclusion threshold to Spearman r < -0.1 or r > 0.4 to include the miRNA in the figure. However, our conclusion that these models are sex-specific still holds, with only *AGO1* selected in both this "alternative male model" and the female-only full model.

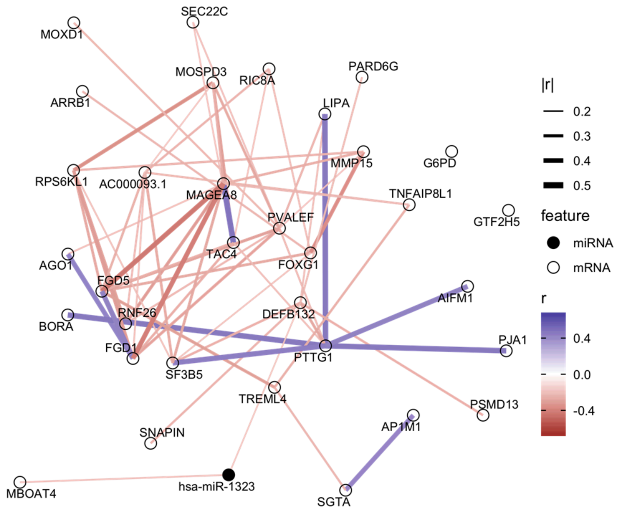

Supplementary Figure S3: Example alternative candidate network in males obtained with null design model, based on hsa-miR-1323.

We also compared the sex-specific models to an all-samples full model, which had 4 miRNAs (hsa-miR-200a-3p, hsa-miR-29a-3p, hsa-miR-30a-5p, hsa-miR-30d-5p) and 85 mRNAs selected based on grid-search tuning, of which 46 genes overlapped with the female-only full model, 6 with the male-only full model, and 41 not present in other models (e.g., *IL4R*, *DCTN1*, *ITGA5*).

Interestingly, these all-sample genes also previously reported associations with allergy and asthma. With the caveat that the results must be interpreted with caution, the network diagram for the all-samples model is recreated below using less stringent edge-filtering thresholds of r < -0.10 or r > 0.40, as pairwise correlations were weaker when not stratifying by sex and would have excluded all miRNAs from the diagram. The all-samples model had an AUC = 0.76, slightly less than both sex-specific models.

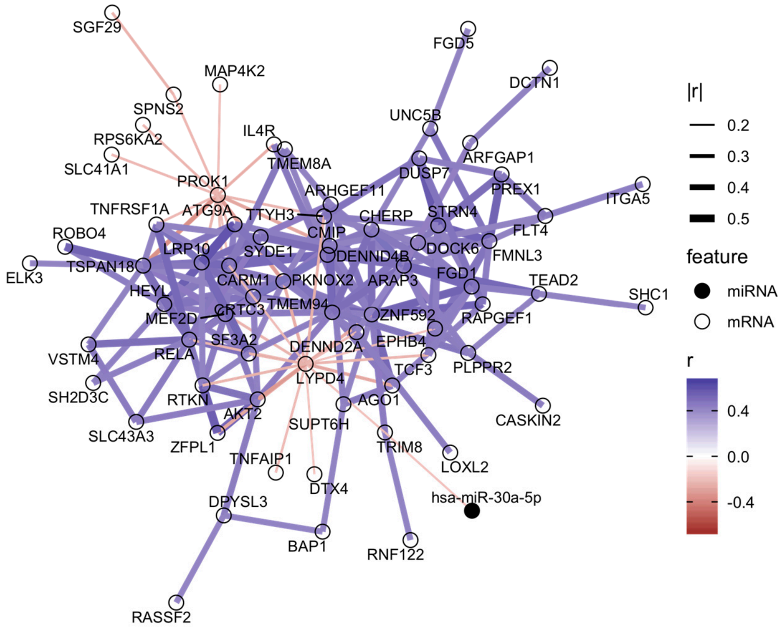

Supplementary Figure S4: All-samples DIABLO full solution.

Lastly, we present a heatmap of variable importance values across the five major models described is shown below, with each row representing one gene, each column one of the described DIABLO models. The scaled mRNA loading value on the DIABLO canonical component is used here as a proxy for variable importance, normalized across models by calculating $v_i = |loading_g| / \Sigma(|loading_g|)$ for all genes g included from the microarray measurement.

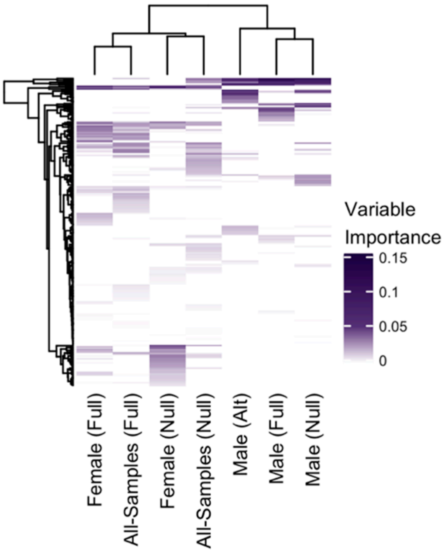

Supplementary Figure S5: Variable importance (normalized model loading; color) of genes (rows) by DIABLO model (columns).

This figure reiterates three notable features.

- The "full" models, which attempt to impose the constraint that features are correlated across miRNA and mRNA modalities, have limited overlap with their respective "null" models in the mRNAs selected. This lends some support that the mRNAs in full models are truly correlated with miRNAs, and thus stronger candidates for miRNA-regulation targets versus just IUS-associated.
- The possible sex-specificity of IUS exposure is supported by the limited overlap between the "male (full)" and "female (full)" DIABLO models. There is also limited overlap between "male (null)" and "female (null)". In contrast, high gene overlap across sexes may have been suggestive of a more universal signature.
- Using the same rationale as point (i), the limited overlap between the all-samples full and all-samples null models may support that the miRNAs selected as being regulators of gene expression. However, the all-samples model must be interpreted with caution due to differences in the strength of the overall miRNA-mRNA correlations by sex (weaker in male samples), biasing "full" models towards female observations.
